# Supplementary material for: Optimizing radionuclide sequestration in anion nanotraps with record pertechnetate sorption
Source: Nat Commun. 2019 Apr 9;10:1646. doi: 10.1038/s41467-019-09630-y (PMC6456584; doi:10.1038/s41467-019-09630-y)
Supplement: Supplementary file 1 — Supplementary Information [file 41467_2019_9630_MOESM1_ESM.pdf]

Supplementary Information

Optimizing Radionuclide Sequestration in Anion Nanotraps with Record Pertechnetate Sorption

Sun et al.

Supplementary Figures

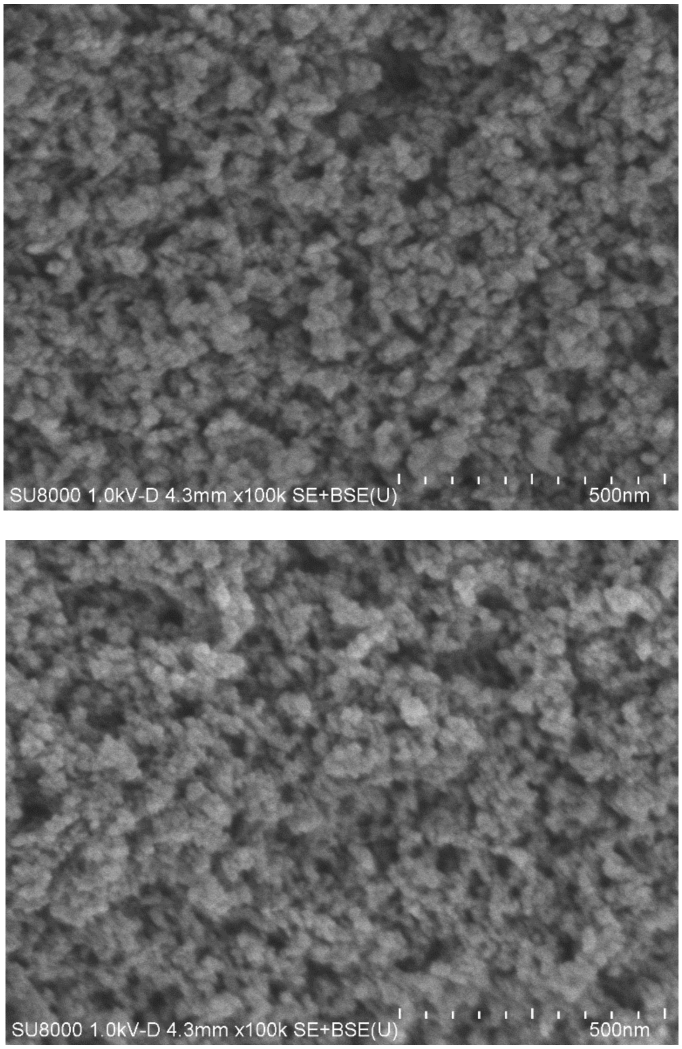

Supplementary Figure 1 | SEM images of POP-*p*N(Me)$_2$Py (top) and PQA-*p*N(Me)$_2$Py-I (bottom).

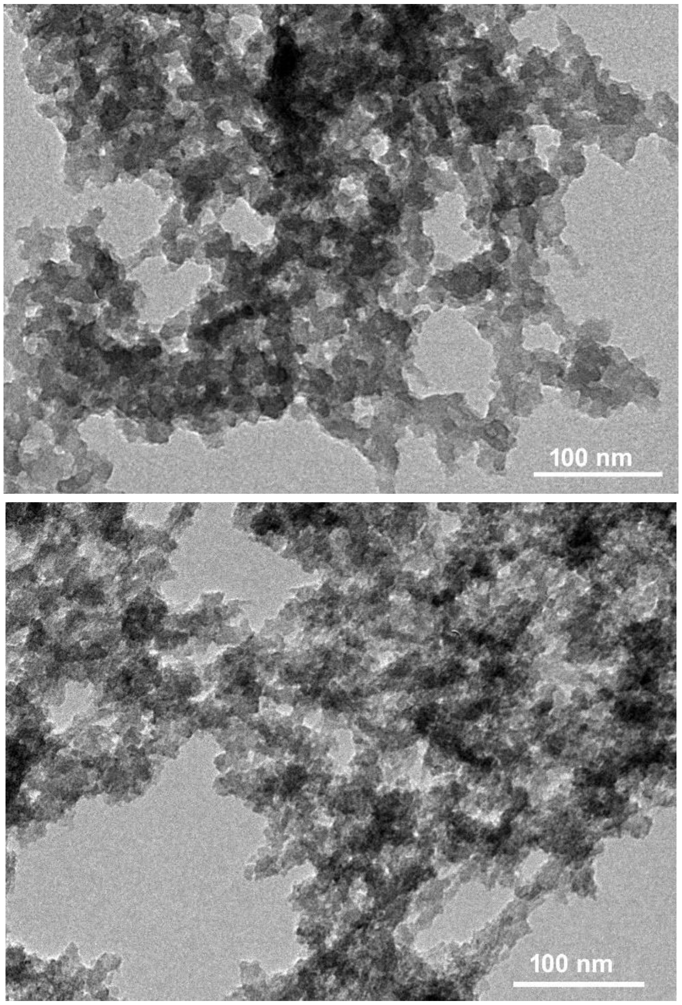

Supplementary Figure 2 | TEM images of POP-*p*N(Me)$_2$Py (top) and PQA-*p*N(Me)$_2$Py-I (bottom).

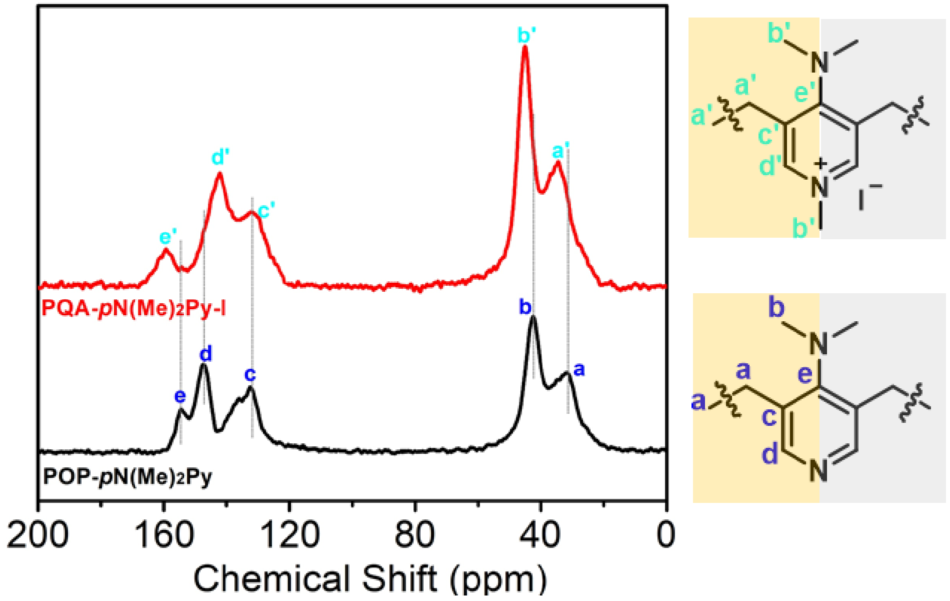

Supplementary Figure 3 | Solid-state ^{13}C NMR spectra of POP-*p*N(Me)$_2$Py and PQA-*p*N(Me)$_2$Py-I.

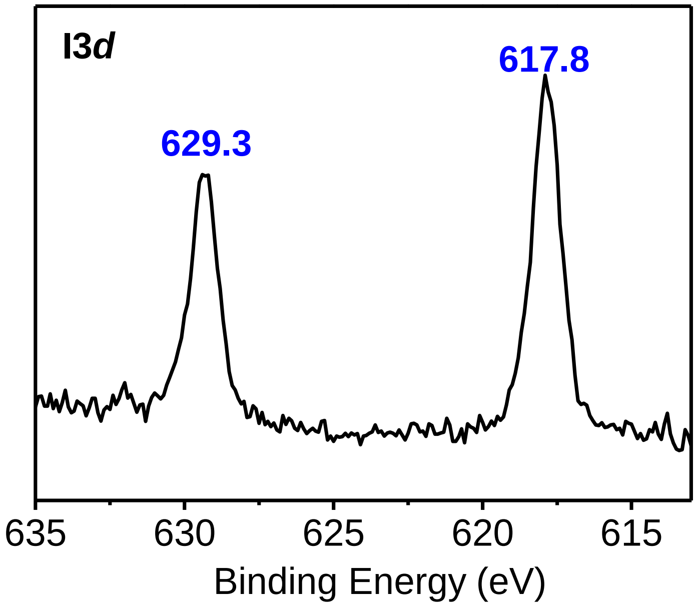

Supplementary Figure 4 | I3*d* XPS spectrum of PQA-*p*N(Me)$_2$Py-I.

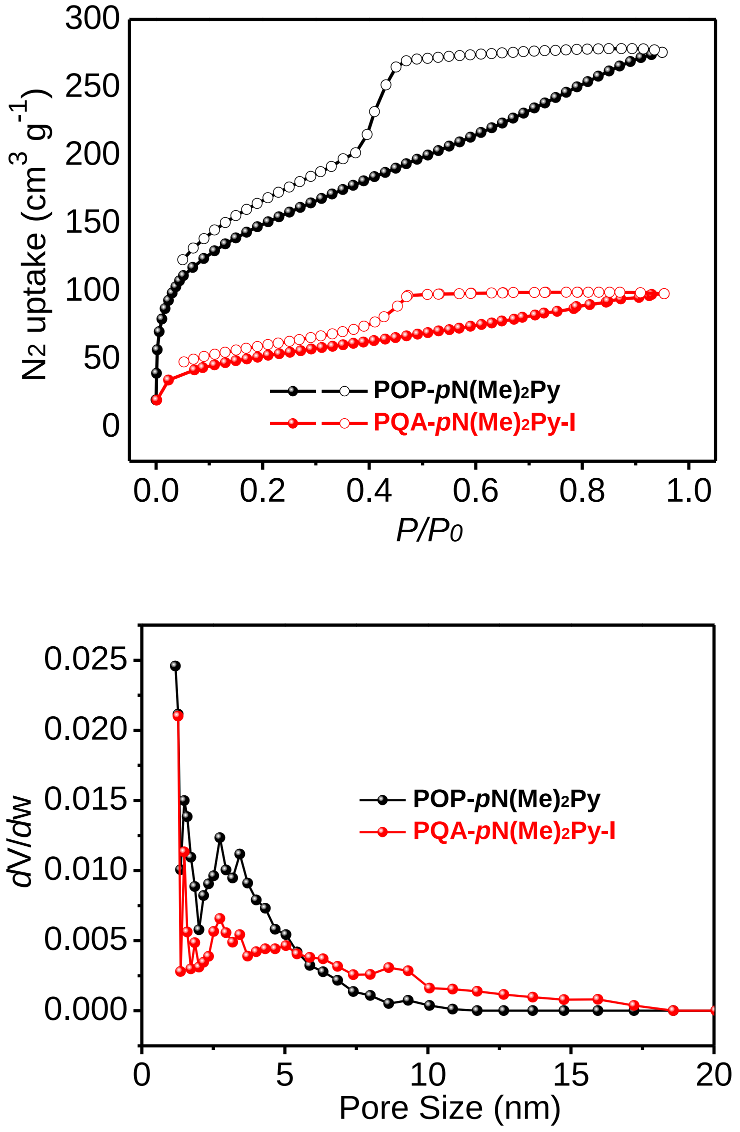

Supplementary Figure 5 | N_2 sorption isotherms for POP-*p*N(Me)$_2$Py and PQA-*p*N(Me)$_2$Py-I collected at 77 K with BET surface areas of 535 and 223 m^2 g^{-1} (Langmuir), respectively (top) and corresponding pore size distribution (bottom).

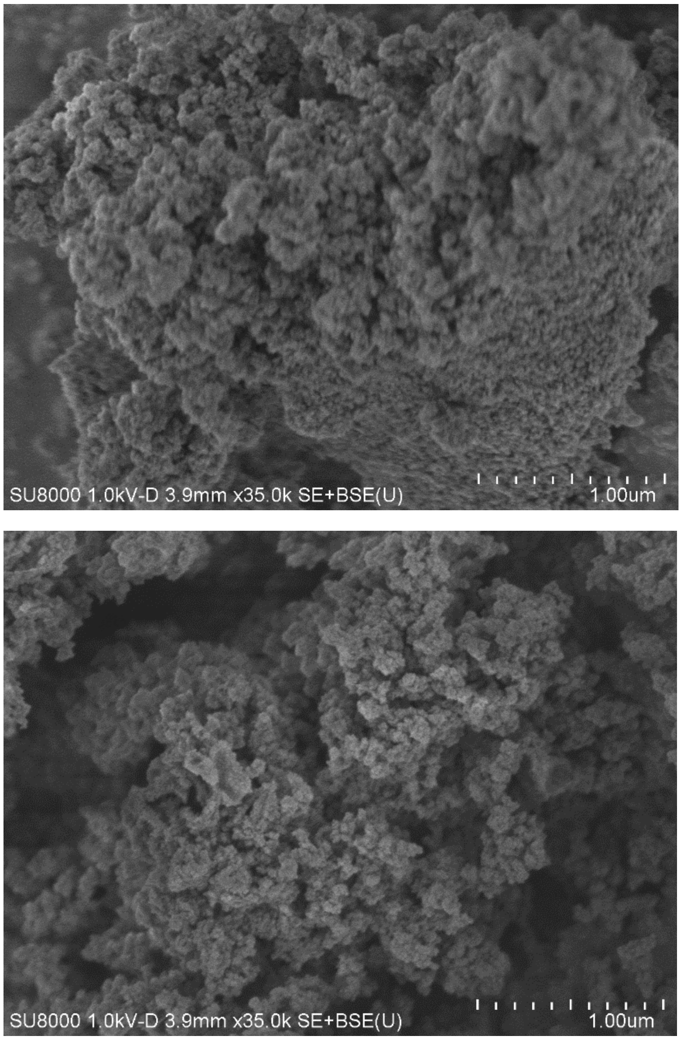

Supplementary Figure 6 | SEM images of POP-Py (top) and PQA-Py-I (bottom).

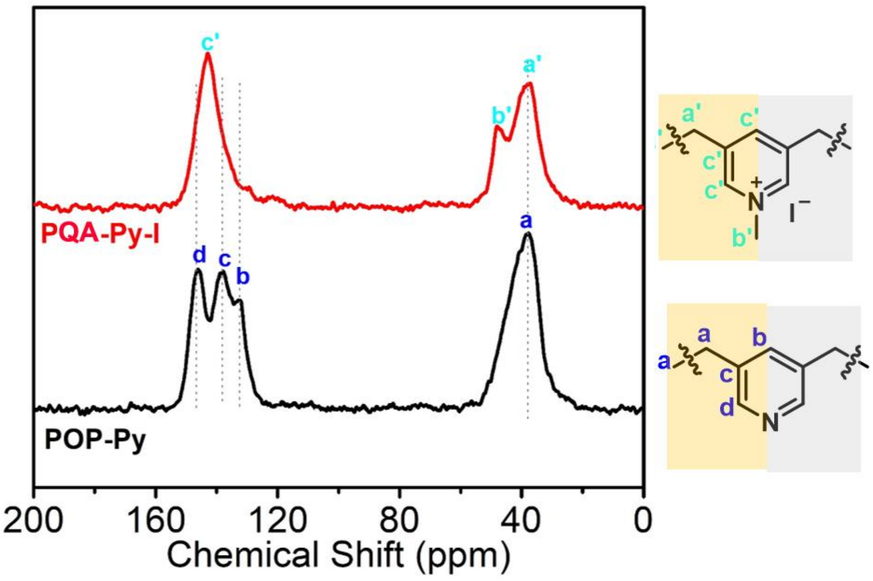

Supplementary Figure 7 | Solid-state ^{13}C NMR spectra of POP-Py and PQA-Py-I.

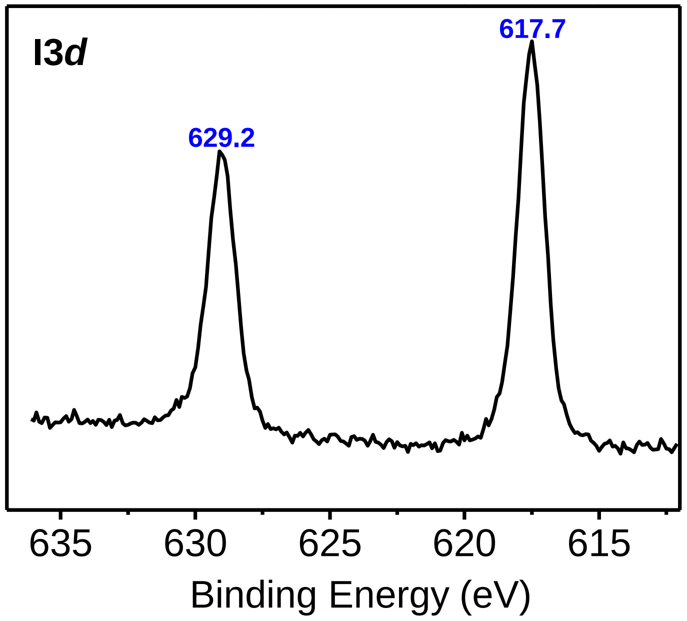

Supplementary Figure 8 | I3*d* XPS spectrum of PQA-Py-I.

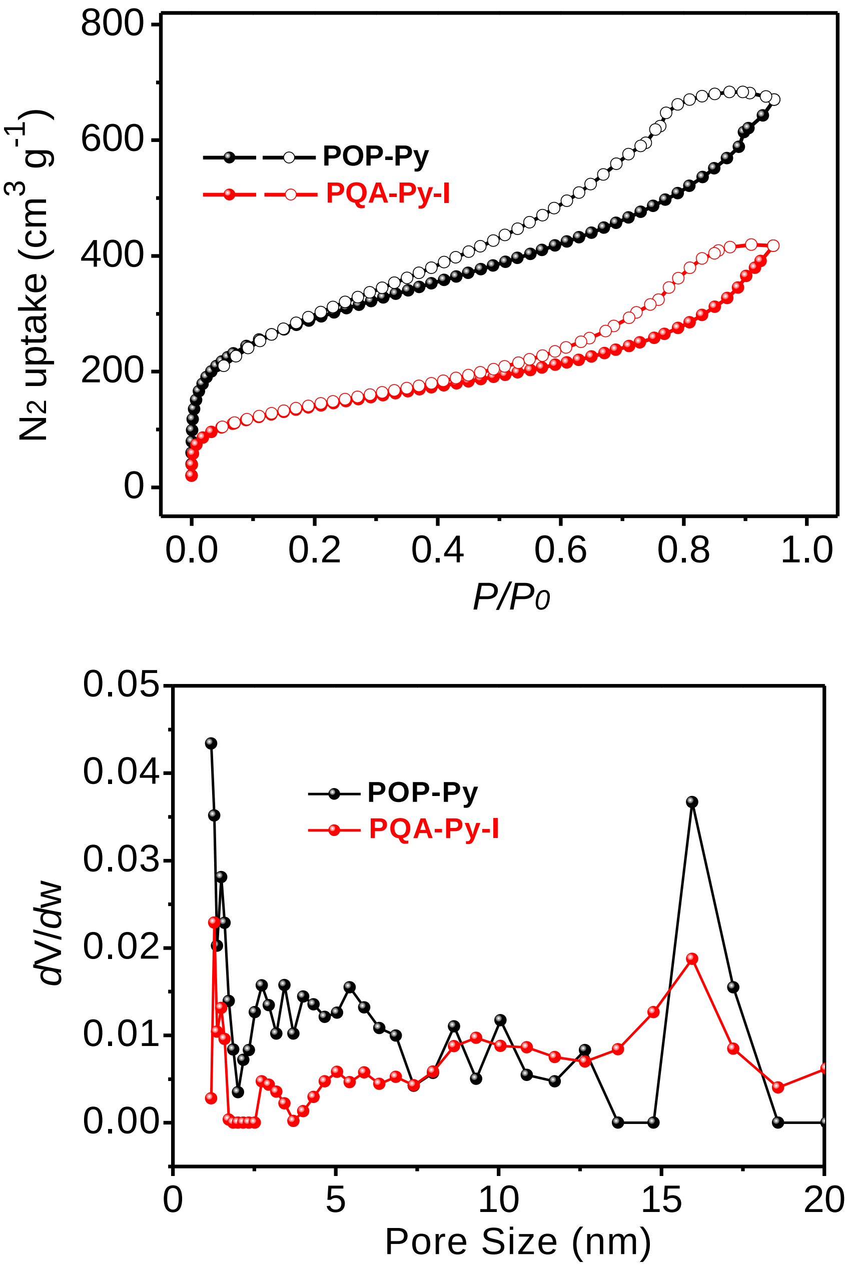

Supplementary Figure 9 | N_2 sorption isotherms for POP-Py and PQA-Py-I collected at 77 K with BET surface areas of 979 and 465 m^2 g^{-1}, respectively (top) and corresponding pore size distribution (bottom).

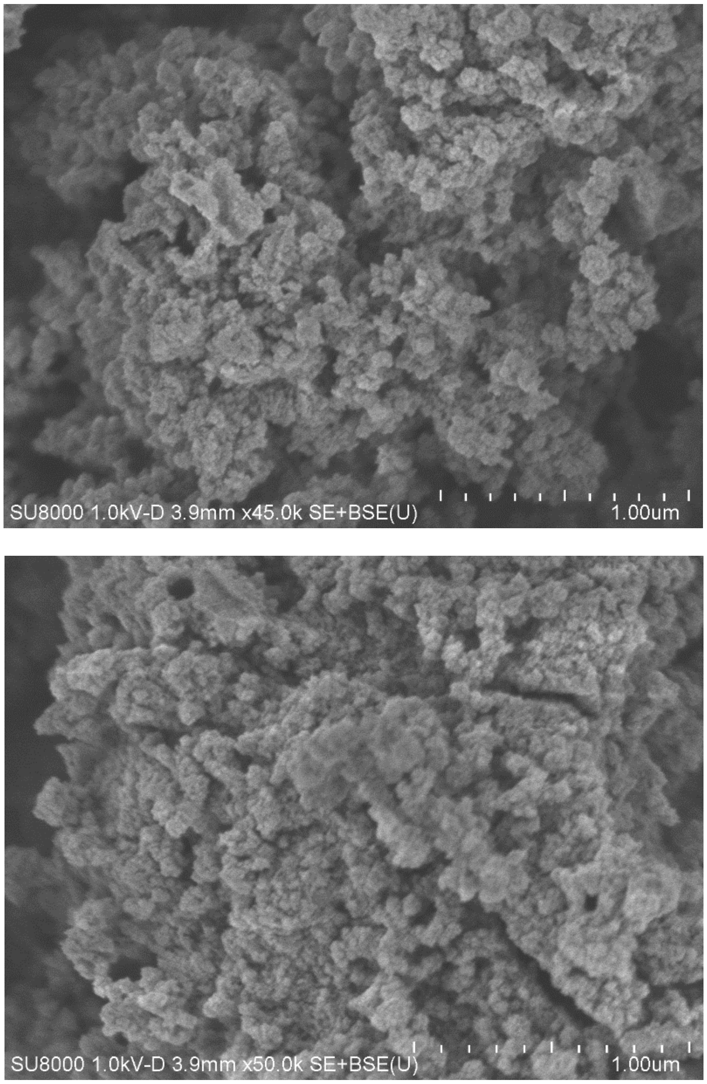

Supplementary Figure 10 | SEM images of POP-*p*NH_2Py (top) and PQA-*p*NH_2Py-I (bottom).

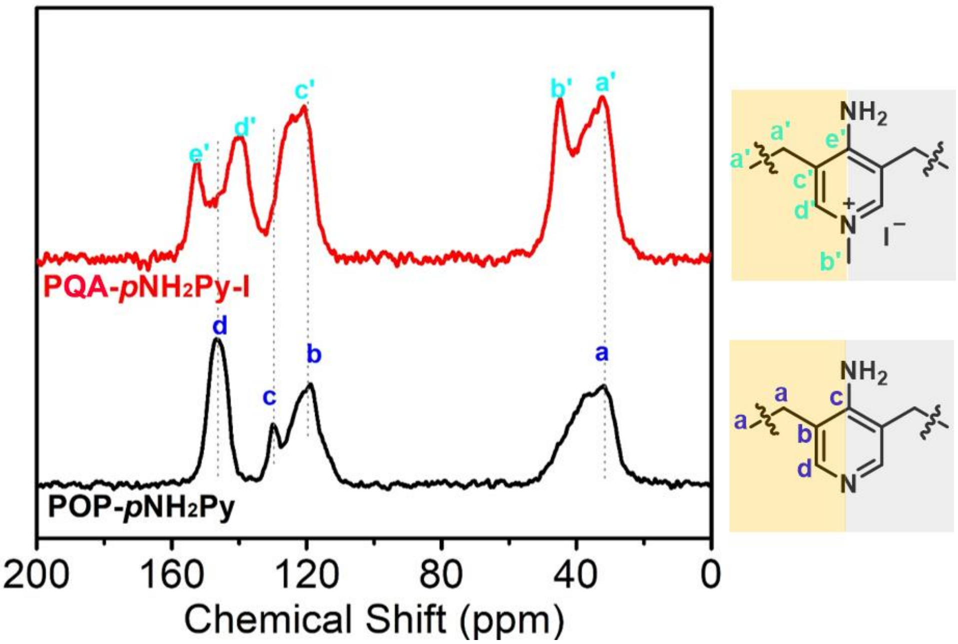

Supplementary Figure 11 | Solid-state ^{13}C NMR spectra of POP-*p*NH_2Py and PQA-*p*NH_2Py-I.

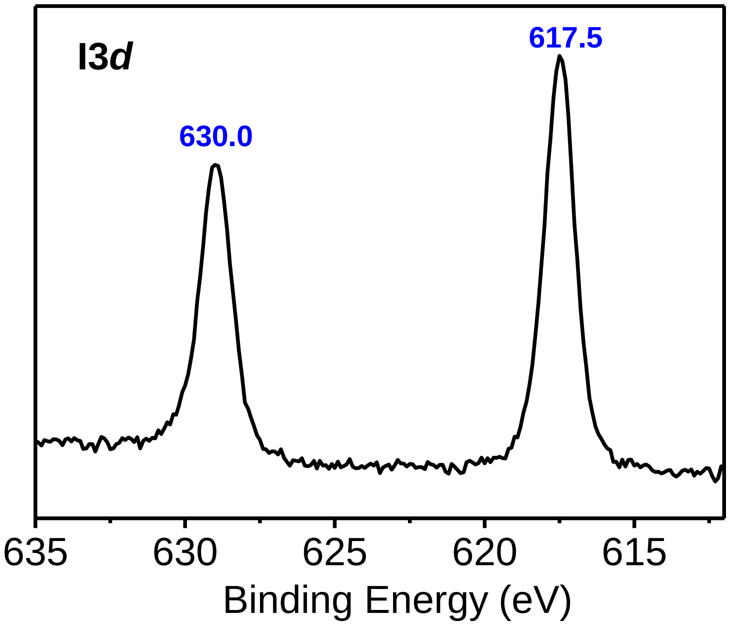

Supplementary Figure 12 | I3*d* XPS spectrum of PQA-*p*NH_2Py-I.

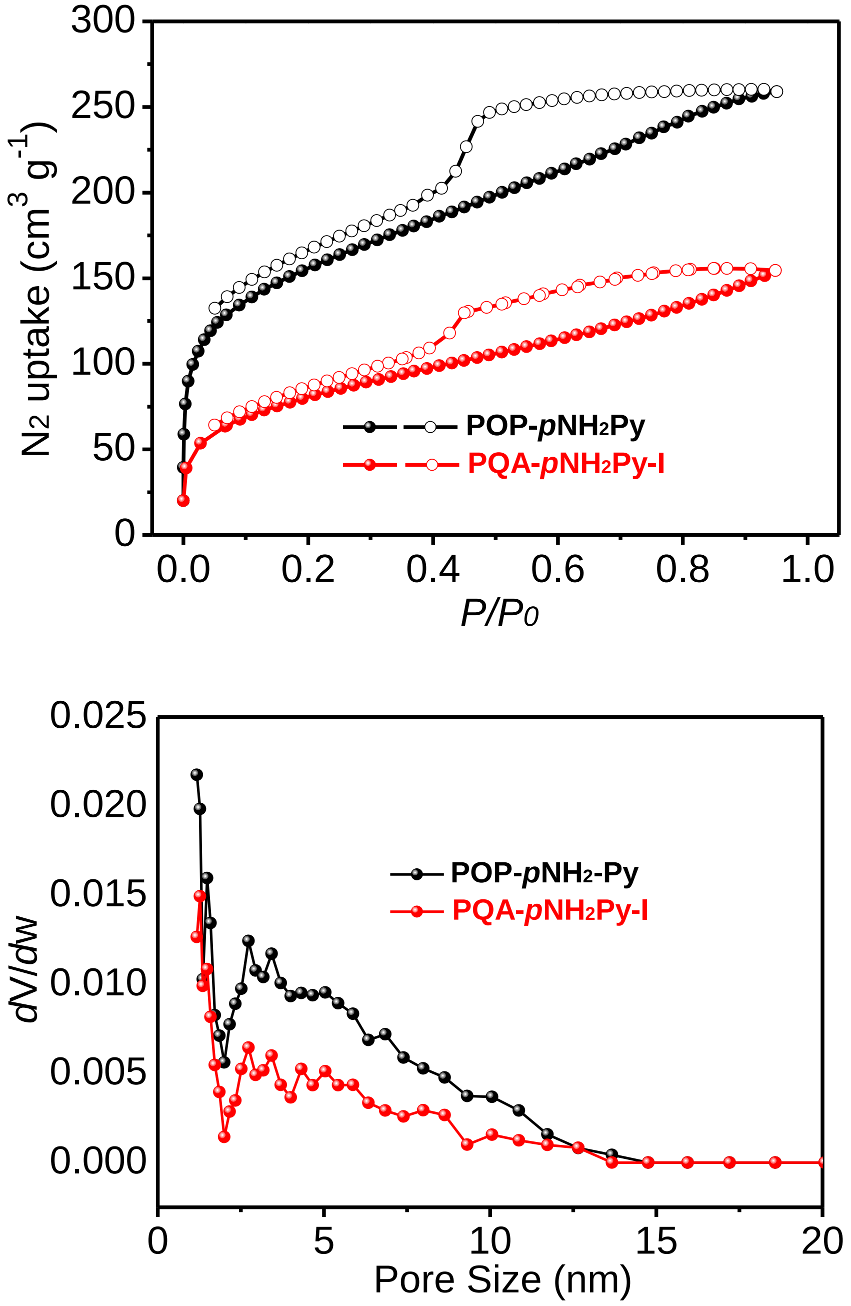

Supplementary Figure 13 | N_2 sorption isotherms for POP-*p*NH_2Py and PQA-*p*NH_2Py-I collected at 77 K with BET surface areas of 536 and 344 m^2 g^{-1}, respectively (top) and corresponding pore size distribution (bottom)..

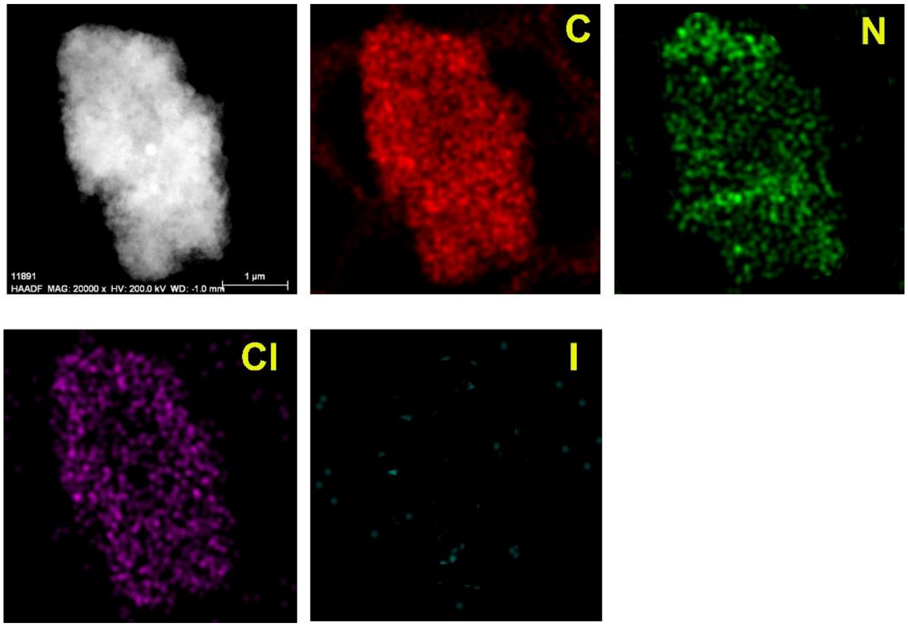

Supplementary Figure 14 | HADDF-STEM and corresponding element mapping of PQA-*p*N(Me)$_2$Py-Cl. The negligibly detected iodine species signal in PQA-*p*N(Me)$_2$Py-Cl indicates the complete ion exchange between I^- and Cl^- ions.

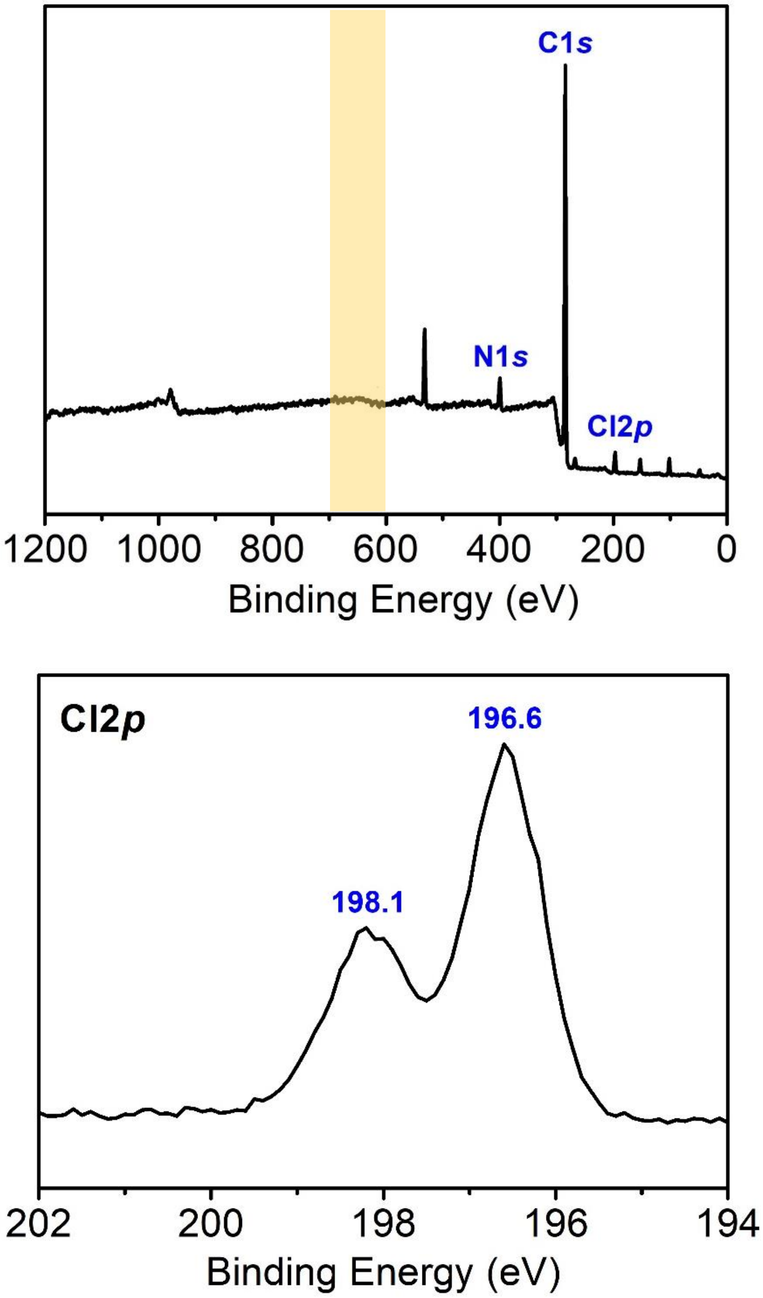

Supplementary Figure 15 | XPS spectra of PQA-*p*N(Me)$_2$Py-Cl. (a) survey spectrum (top) and (b) Cl2*p* spectrum (bottom). The disappearance of peaks in the range of 610 to 650 eV related to iodine species and the concomitant emergence of strong peaks at 196.6 eV and 198.1 eV attributable to the chloride species, indicative the complete ion exchange between Cl^- and I^- ions.

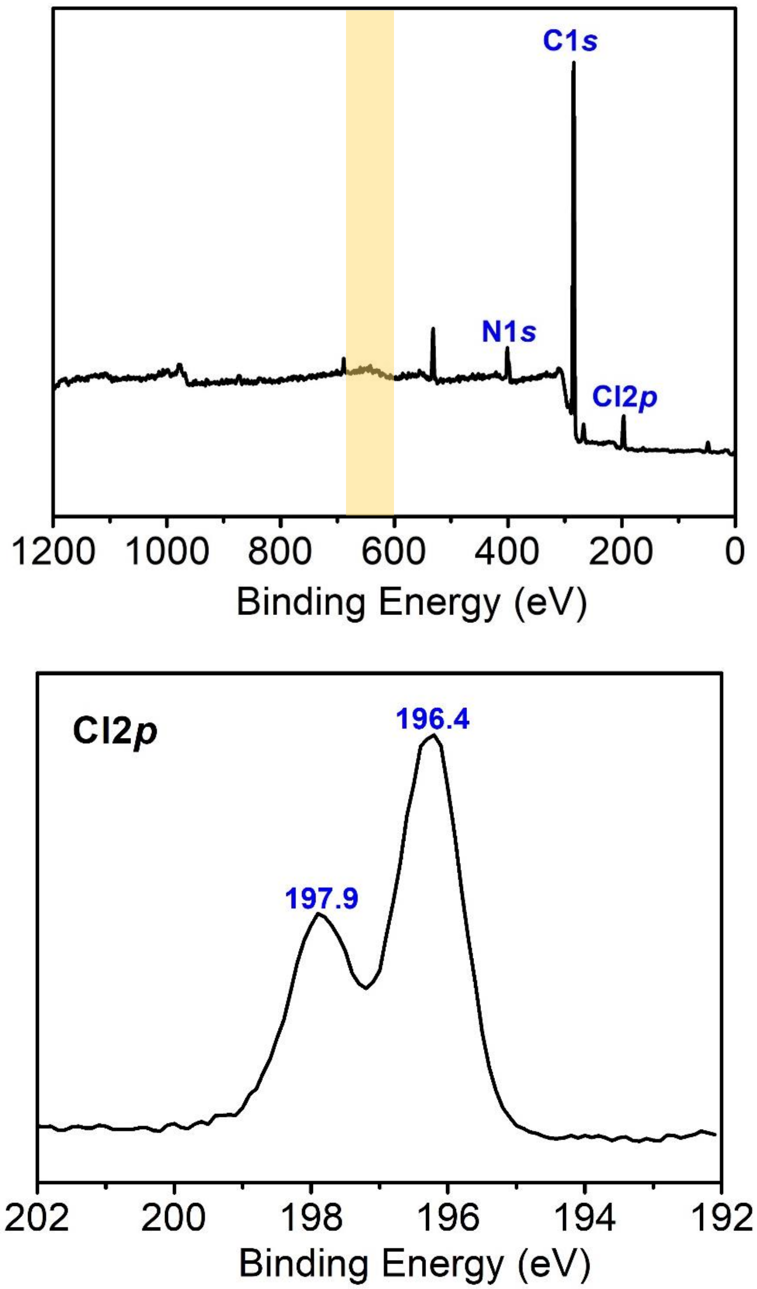

Supplementary Figure 16 | XPS spectra of PQA-Py-Cl. (a) survey spectrum (top) and (b) Cl2*p* spectrum (bottom). The disappearance of peaks in the range of 610 to 650 eV related to iodine species and the concomitant emergence of strong peaks at 196.4 eV and 197.9 eV attributable to the chloride species, indicative the complete ion exchange between Cl^- and I^- ions.

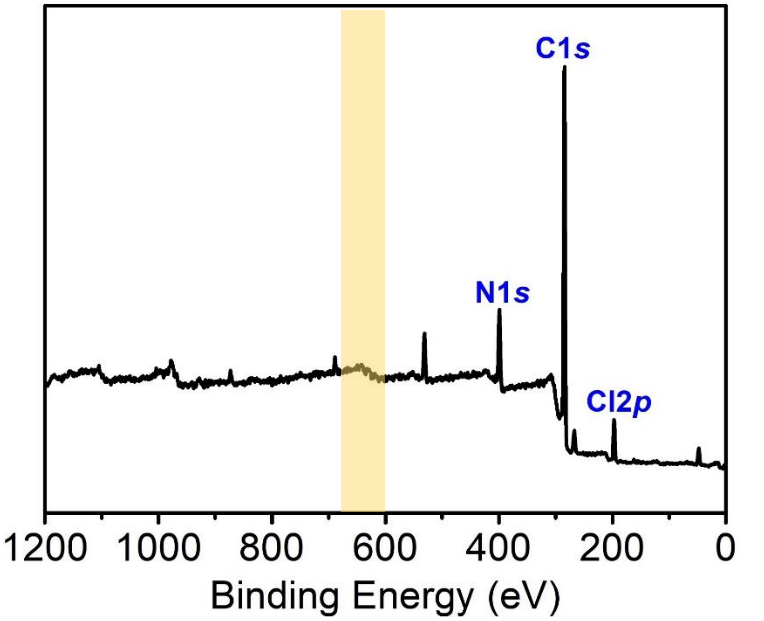

Supplementary Figure 17 | XPS spectra of PQA-*p*NH_2Py-Cl. (a) survey spectrum (top) and (b) Cl2*p* spectrum (bottom). The disappearance of peaks in the range of 610 to 650 eV related to iodine species and the concomitant emergence of strong peaks at 196.4 eV and 197.9 eV attributable to the chloride species, indicative the complete ion exchange between Cl^- and I^- ions.

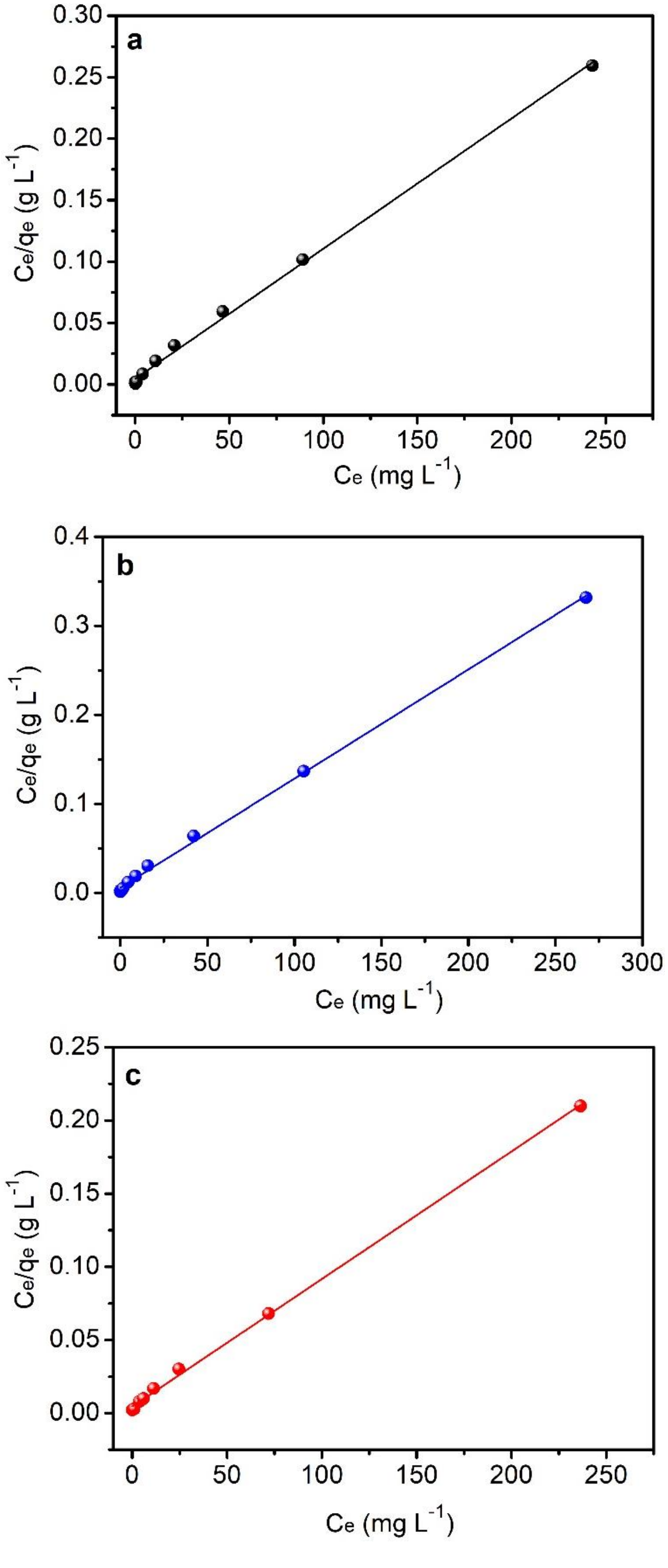

Supplementary Figure 18 | Linear regression by fitting the equilibrium data with the Langmuir adsorption model shown in Fig. 2a of the main text. (a) PQA-Py-Cl, (b) PQA-*p*NH_2Py-Cl, and (c) PQA-*p*$N(Me)_2$-Cl.

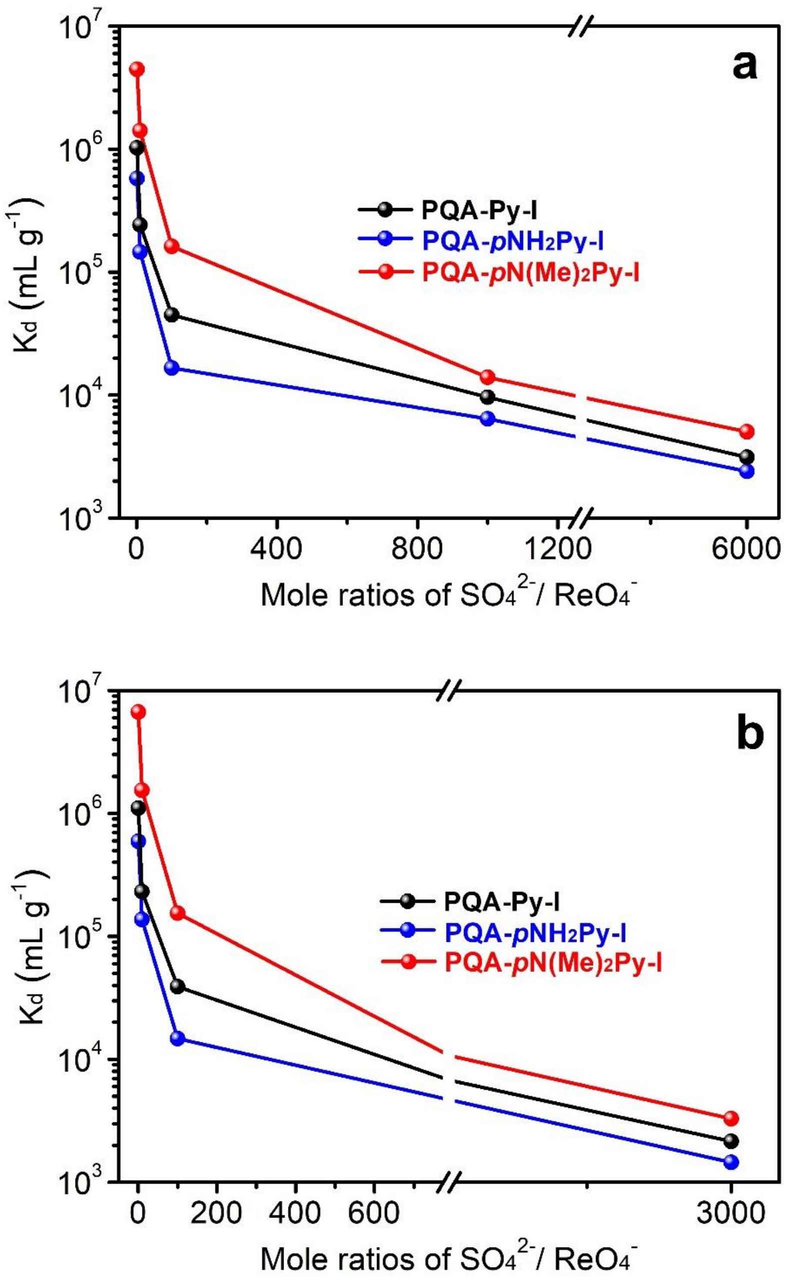

Supplementary Figure 19 | Effect of competing (a) SO_4^{2-} and (b) NO_3^- anions on the binding affinity of various adsorbents toward ReO_4^-.

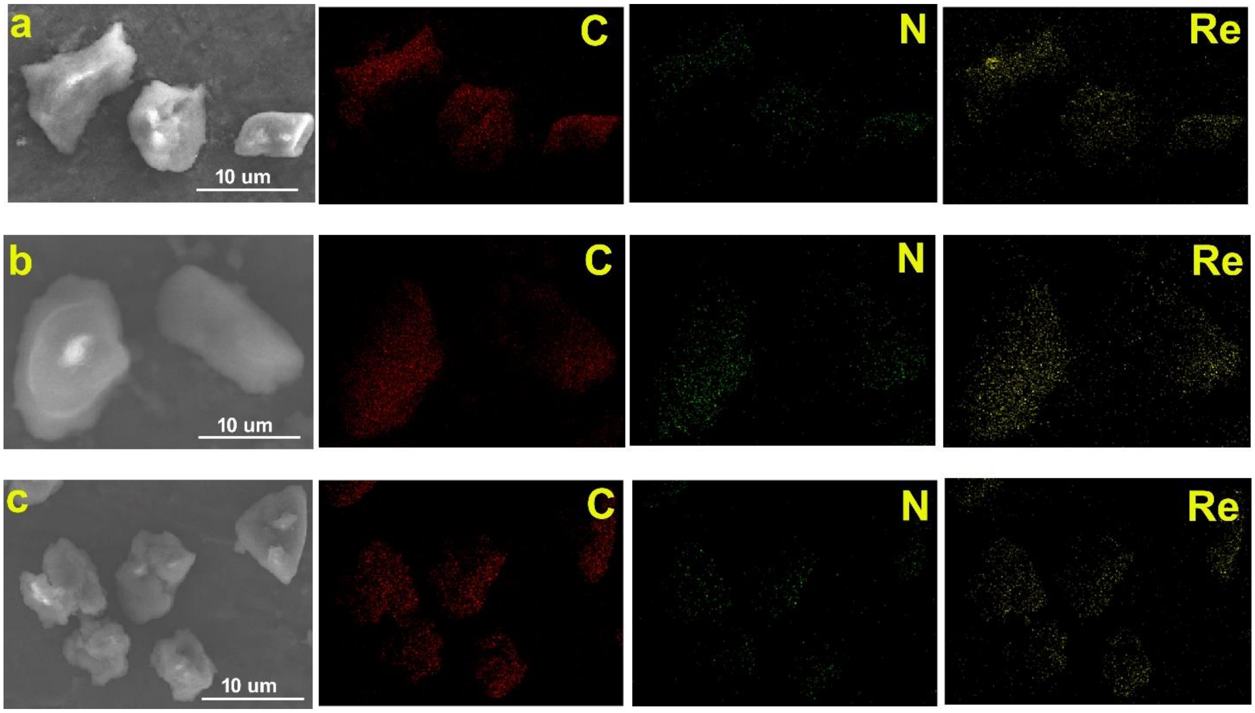

Supplementary Figure 20 | SEM images and corresponding EDX mapping. (a) Re@PQA-Py-Cl, (b) Re@PQA-*p*NH_2Py-Cl, and (c) Re@PQA-*p*N$(Me)_2$Py-Cl.

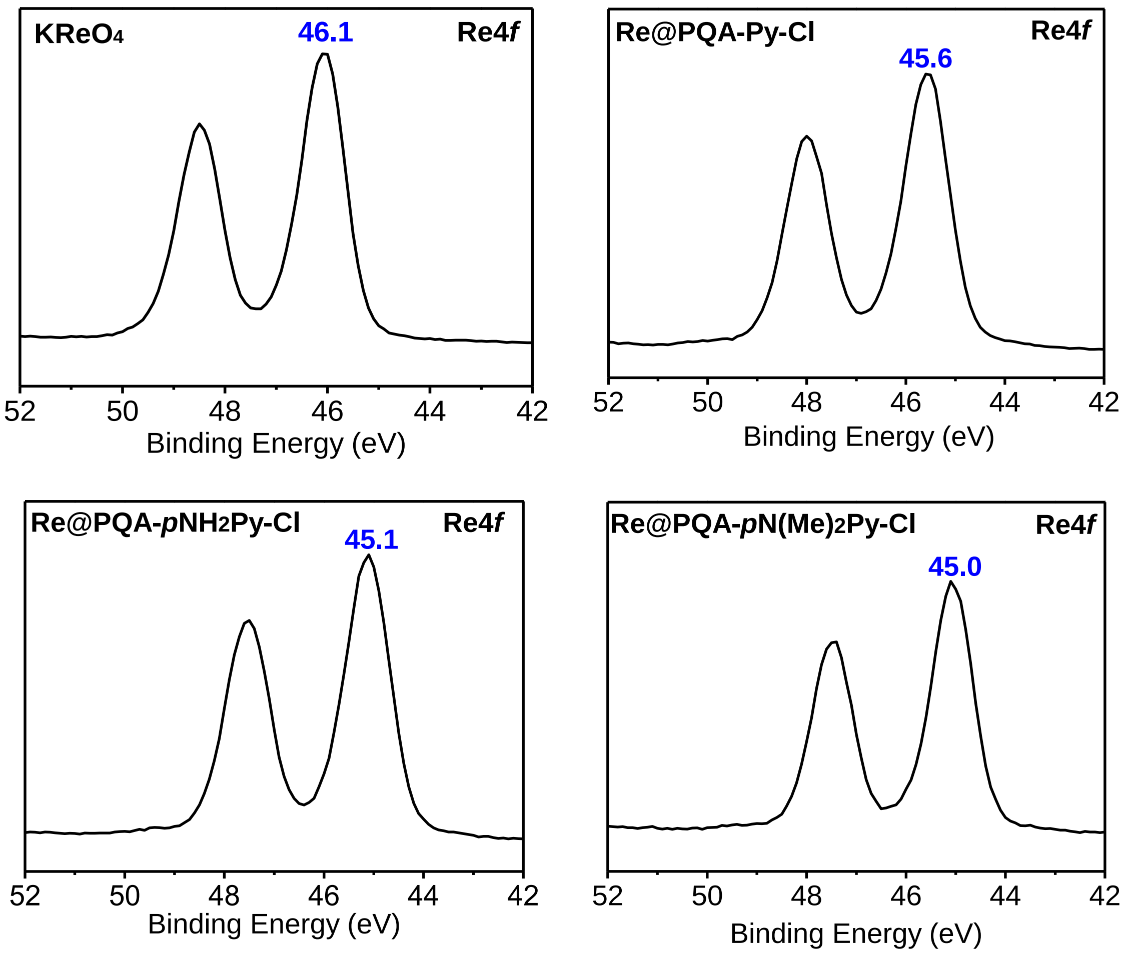

Supplementary Figure 21 | Re4*f* XPS spectra of $KReO_4$, Re@PQA-Py-Cl, Re@PQA-*p*NH_2Py-Cl, and Re@PQA-*p*N$(Me)_2$Py-Cl, respectively.

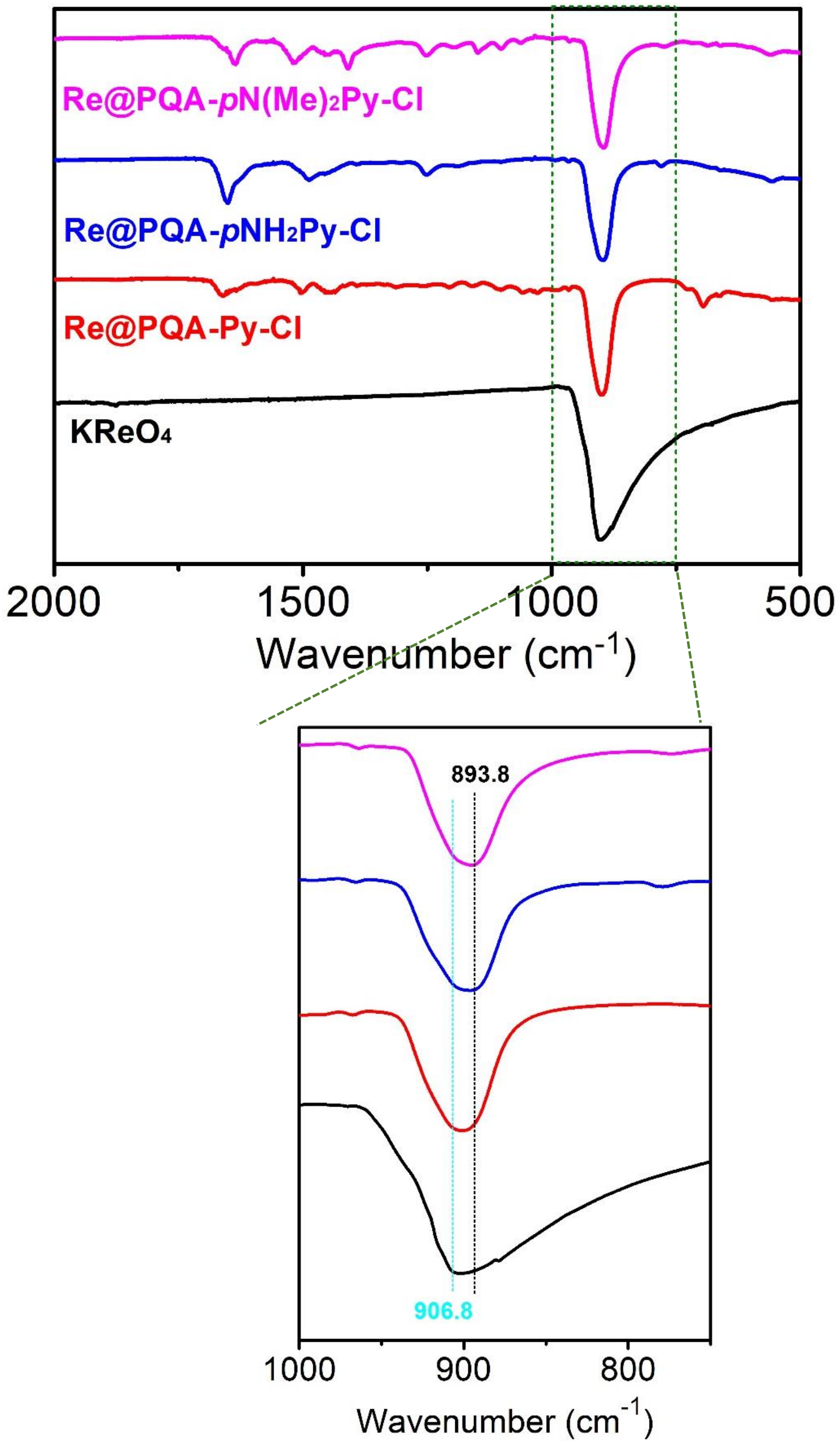

Supplementary Figure 22 | IR spectra of $KReO_4$, Re@PQA-Py-Cl, Re@PQA-*p*NH_2Py-Cl, and Re@PQA-*p*N$(Me)_2$Py-Cl, respectively.

.

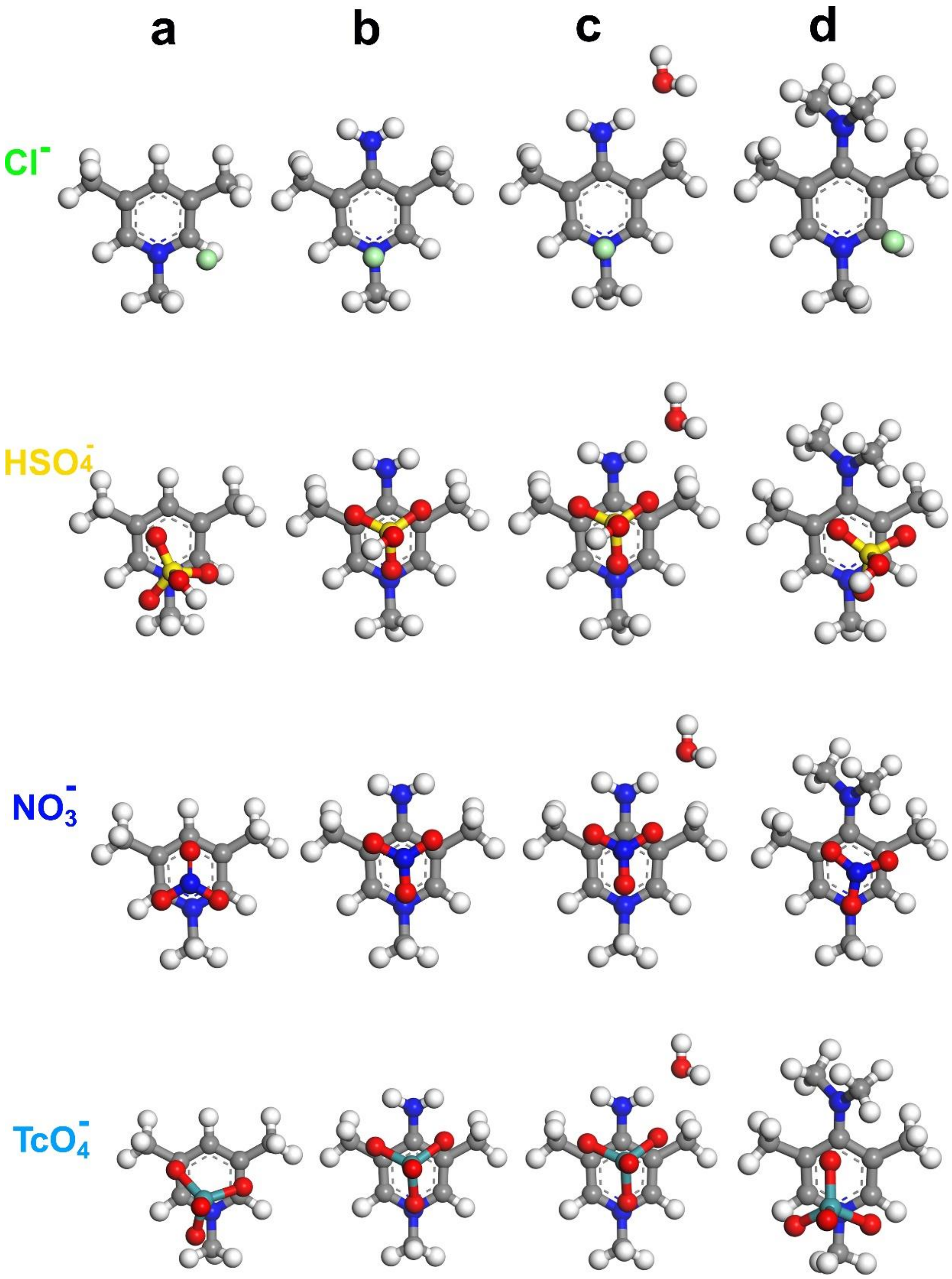

Supplementary Figure 23 | Top-down view of minimum energy anion binding positions of various anions with building blocks of (a) PQA-Py-Cl, (b,c) PQA-*p*NH_2Py-Cl, and (d) PQA-*p*N$(Me)_2$Py-Cl for all 12 calculations. Grey, blue, red, cyan, green, and orange atoms are carbon, nitrogen, oxygen, technetium, chlorine, and sulfur atoms, respectively.

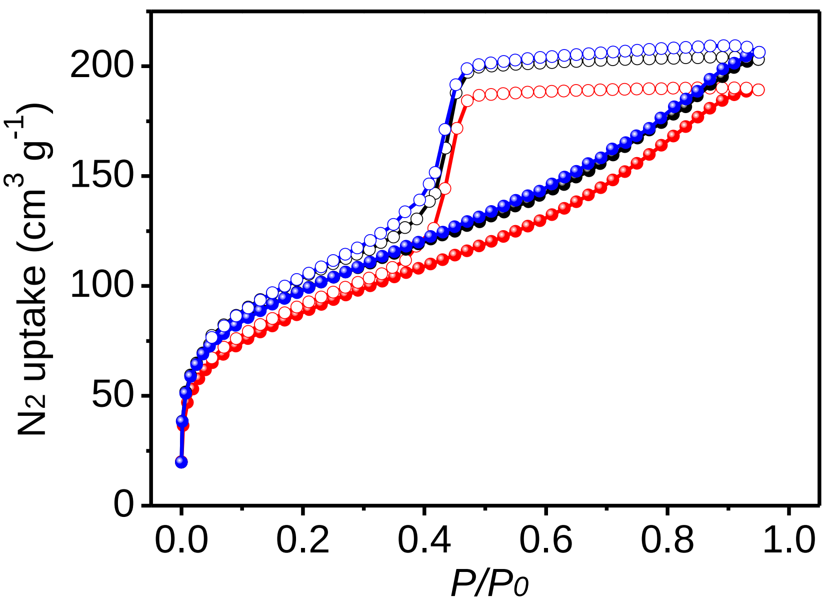

Supplementary Figure 24 | N_2 sorption isotherms collected at 77 K for PQA-*p*N(Me)$_2$Py-Cl (black) and that after being soaked in 12 M HCl (blue) or 2 M NaOH (red) in saturated NaCl aqueous solution for one week. The BET surface areas were calculated to be 328, 330, and 296 $m^2 g^{-1}$, respectively, indicative of its excellent stability towards a wide range acid-base environments.

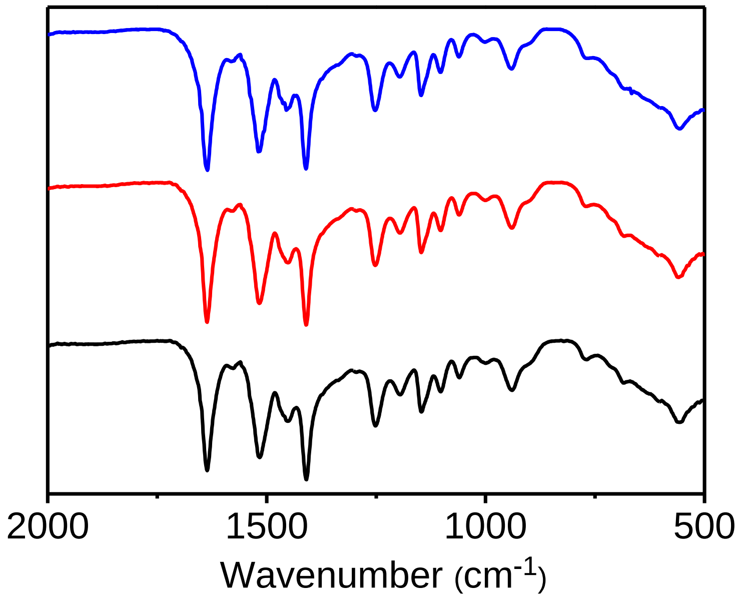

Supplementary Figure 25 | IR spectra of PQA-*p*N(Me)$_2$Py-Cl (black) before and after being irradiated with varied doses of β-rays (red) or γ-rays (blue).

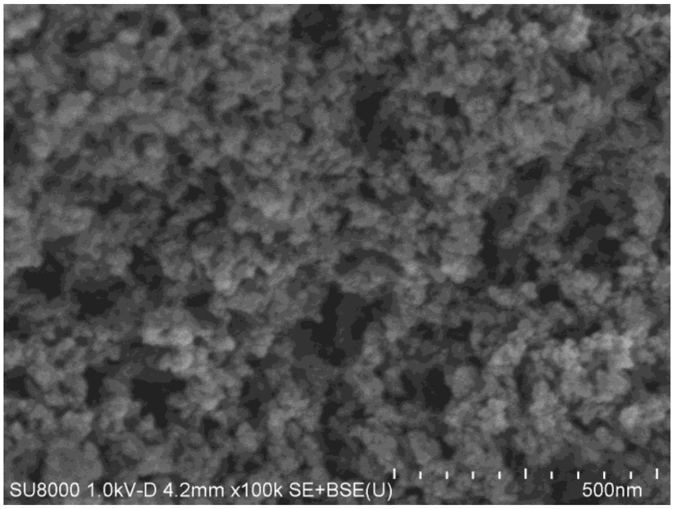

Supplementary Figure 26 | SEM image for PQA-*p*N(Me)$_2$Py-Cl after being irradiated with a dose of 1000 kGy of β-rays.

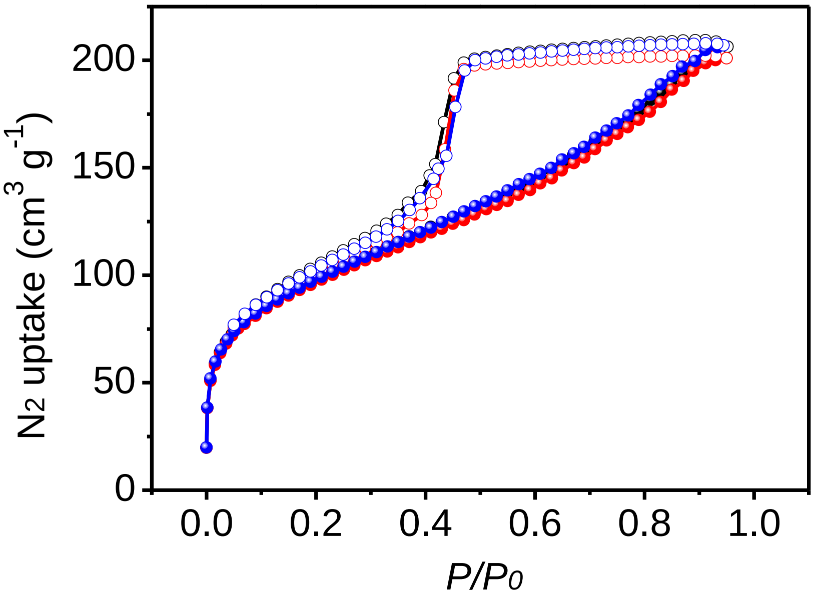

Supplementary Figure 27 | N_2 sorption isotherms collected at 77 K for PQA-*p*N(Me)$_2$Py-Cl after being irradiated with a dose of 1000 kGy of β-rays (red) or γ-rays (blue). The BET surface areas were calculated to be 328, and 324 m^2 g^{-1}, respectively, with negligible change in comparison with that of the pristine sample (328 m^2 g^{-1}, black), indicative of its radioactive stability.

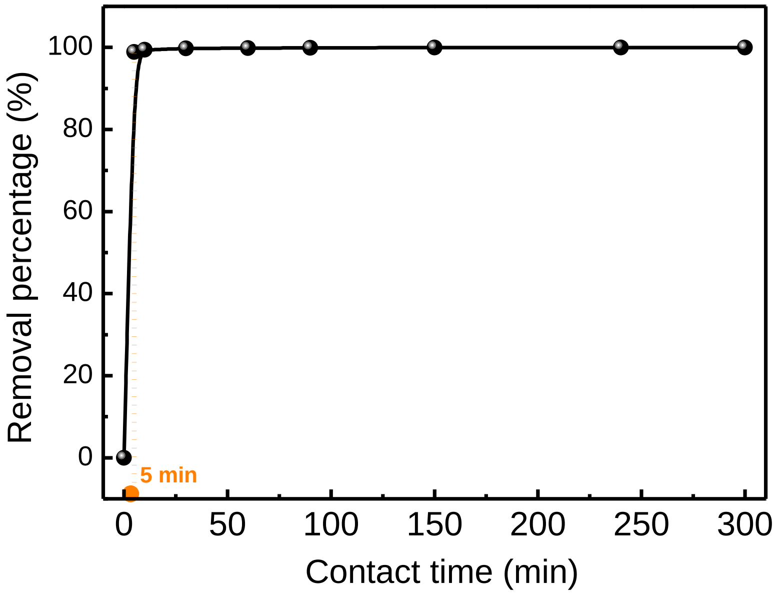

Supplementary Figure 28 | Sorption kinetics of TcO_4^- by PQA-*p*N(Me)$_2$Py-Cl from aqueous solution with an initial concentration of 28 ppm and at a phase ratio (V/m) of 1000 mL g^{-1}.

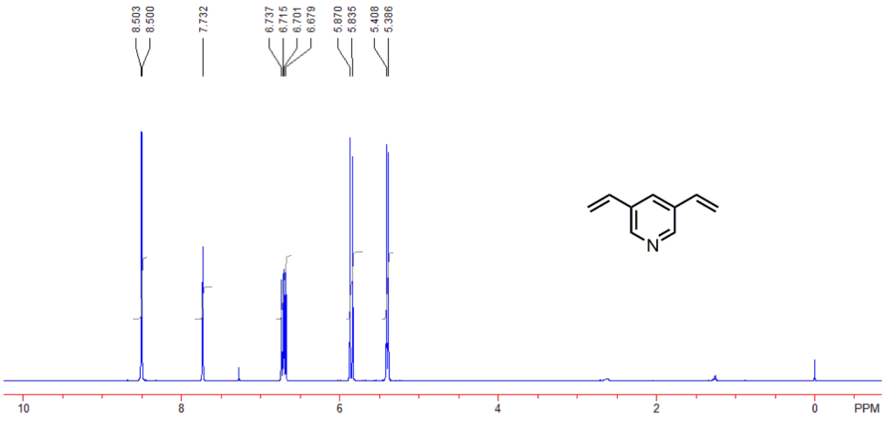
8.503
8.500
7.732
6.737
6.715
6.701
6.679
5.870
5.835
5.408
5.386
N
10
8
6
4
2
0
PPM

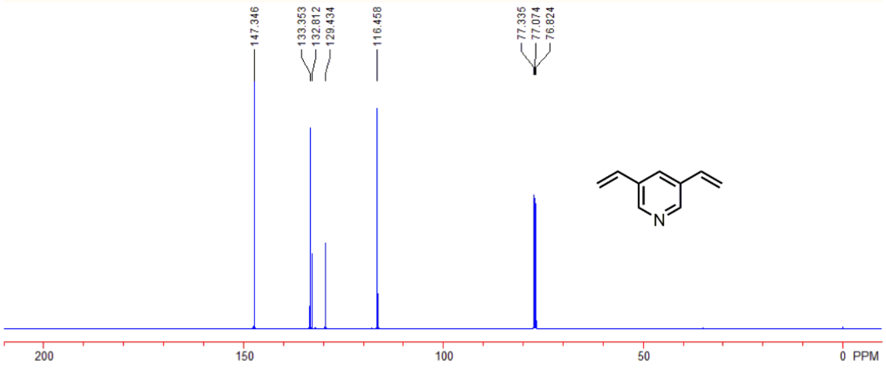
147.346
133.353
132.812
129.434
116.458
77.335
77.074
76.824
N
200
150
100
50
0 PPM

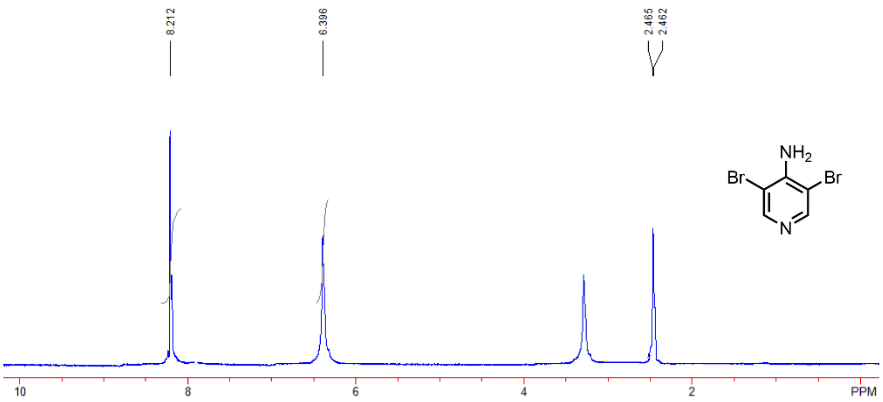
8.212
6.396
2.465
2.462
NH_2
Br
Br
N
10
8
6
4
2
PPM

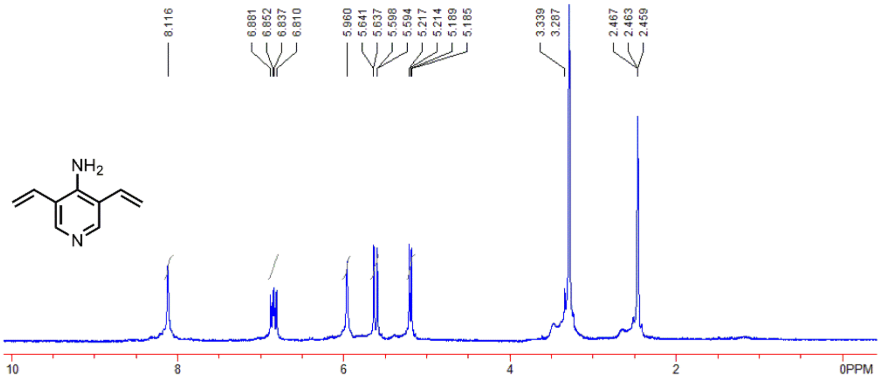
8.116
6.881
6.852
6.837
6.810
5.960
5.641
5.637
5.598
5.594
5.217
5.214
5.189
5.185
3.339
3.287
2.467
2.463
2.459
NH_2
N
10
8
6
4
2
0PPM

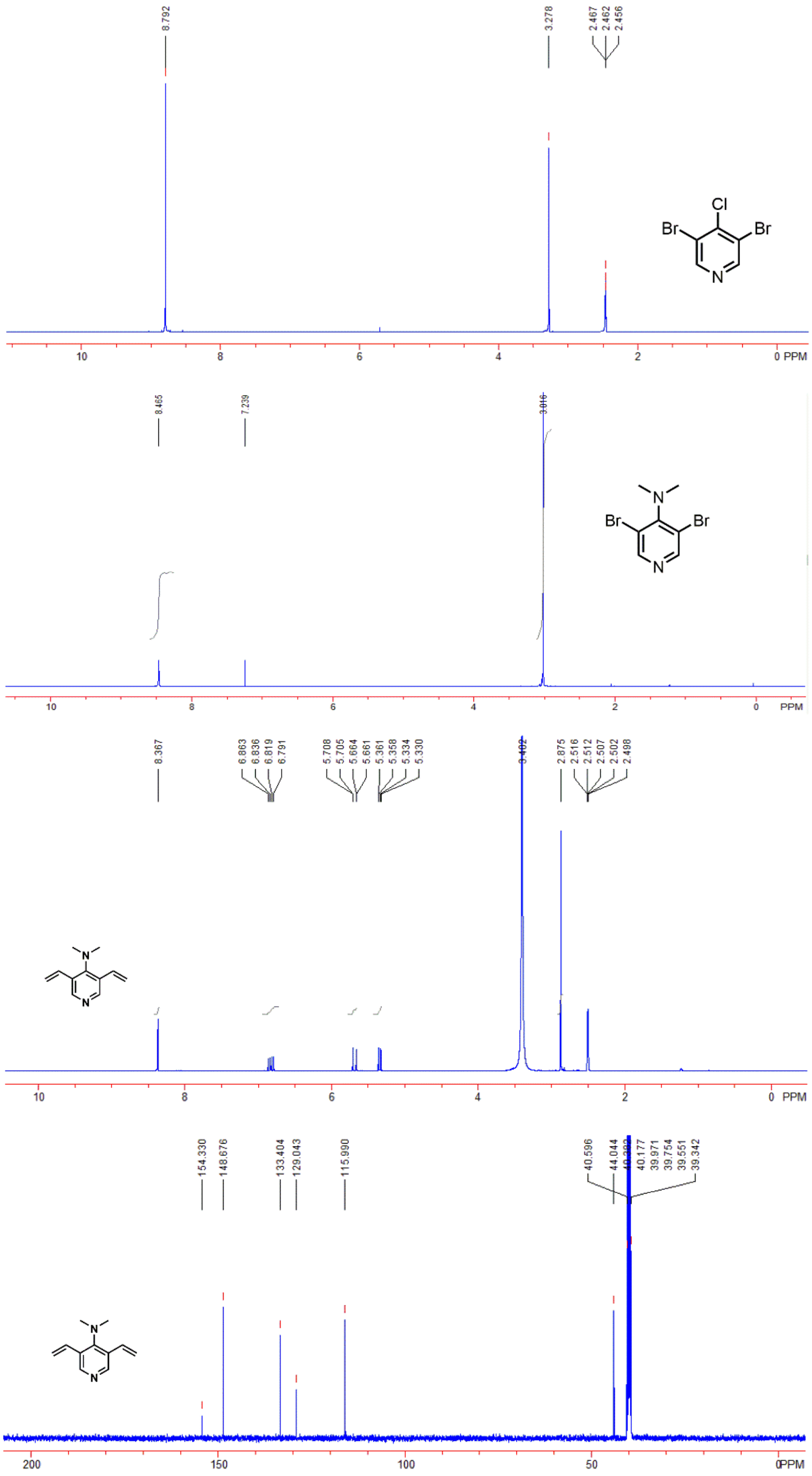

Supplementary Figure 29 | Liquid NMR spectra of various compounds.

Supplementary Tables

Supplementary Table 1 | The iodine species content in various samples evaluated by elemental analysis.

Sample	Theoretical values (wt.%)	Elemental analysis (wt.%)
PQA-Py-I	46.5	45.6
PQA-*p*NH_2Py-I	44.1	41.5
PQA-*p*N(Me_2)Py-I	40.2	38.3

Supplementary Table 2 | ReO_4^- uptake capacities over various cationic materials.

Category	Sorbents	Capacity (mg g^{-1})
Inorganic materials	Nano-SiO_2 [1]	4.94
	$Yb_3O(OH)_6Cl$ [1]	48.6
	NDTB-1 [2]	162.2
Composites	ZrO_2@rGO [3]	43.55
	GO-DEA-DIBA [4]	140.82
Resin	PP-g-VP [5]	113
	PS-g-4VP-IE [6]	252
	D318 [7]	351
	4-ATR [8]	354
	R_2SO_4 [9]	462
	Purolite A532E [10]	446
	Purolite A532E [11]	706
MOF	SBN [10]	786
	SLUG-21 [12]	602
	UiO-66-NH_3^+ [13]	159
	SCU-100 [11]	541
	SCU-101 [14]	217
	NU-1000 [15]	210
POPs	PAF-1-F [16]	420
	SCU-CPN-1 [17]	990
	PQA-pN(Me)$_2$Py-Cl (this work)	1127

Supplementary Table 3 | The chloride species content in various samples evaluated by elemental analysis.

Sample	Theoretical values (wt.%) [a]	Elemental analysis (wt.%)
PQA-Py-Cl	12.7	12.5
PQA-*p*NH_2Py-Cl	11.6	11.1
PQA-*p*N(Me_2)Py-Cl	10.7	10.3

[a] calculated based on the iodine species obtained from elemental analysis.

Supplementary Table 4 | Binding free energy calculation.

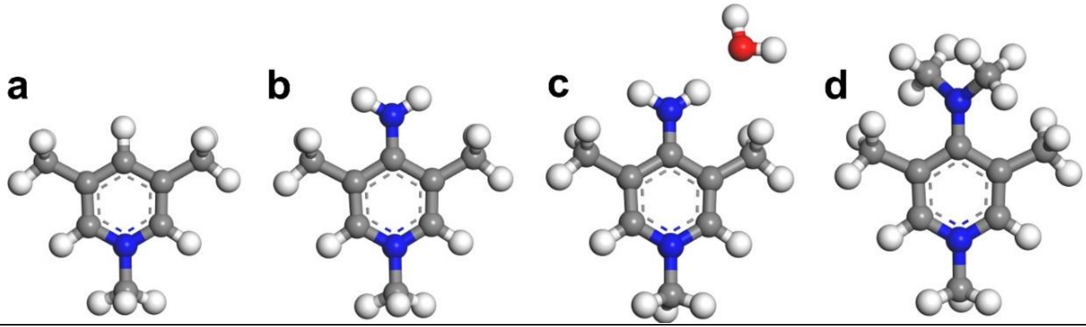

ΔG^0	**a**	**b**	**c**	**d**
Cl^-	-90.3	-79.6	-75.0	-83.2
TcO_4^-	-75.9	-73.4	-69.0	-74.0
HSO_4^-	-81.9	-78.1	-73.1	-77.0
NO_3^-	-83.0	-78.1	-72.8	-79.0
Solvation Free Energy				
Cl^-	-80.0	**a**	-31.8	
TcO_4^-	-67.5	**b**	-31.7	
HSO_4^-	-75.9	**c**	-29.6	
NO_3^-	-78.2	**d**	-27.2	
$\Delta\Delta G_{solv}$	**a**	**b**	**c**	**d**
Cl^-	105.5	93.6	89.7	99.7
TcO_4^-	86.3	85.9	83.0	84.4
HSO_4^-	92.5	91.1	88.0	89.6
NO_3^-	95.6	92.7	88.9	91.8
ΔG_{exch}				
Cl^-	15.2	14.0	14.8	16.5
TcO_4^-	10.4	12.5	14.0	10.4
HSO_4^-	10.6	13.0	14.9	12.6
NO_3^-	12.6	14.6	16.1	12.7

$\Delta\Delta G_{exch}$ (Relative to Cl^-)				
TcO_4^-	-4.8	-1.5	-0.7	-6.1
HSO_4^-	-4.6	-1.0	0.1	-3.9
NO_3^-	-2.6	0.6	1.3	-3.8
All energies in kcal mol^{-1}				

The top rows show quantum-mechanical binding free energies for each complex relative to the dissociated molecules using a 1M standard state. The second set of rows tabulate the (RISM3D-KH) solvation free energies computed for the dissociated molecules and the very next set of rows gives the solvation free energies of the complexes. Rows labeled G_{solv} give the part of the solvation free energy due only to solvation effects. The third set of rows gives the ion estimated binding constants for attachment of anions to free (+1 charged) monomers in solution. The final set of rows provide the free energy of ion exchange into the polymer phase.

Supplementary Table 5 | Composition of simulated Hanford LAW melter recycle stream.

Anion	Concentration [M]	Anion:TCO_4^- Mole Ratio
TcO_4^-	1.94 x 10^{-4}	1.0
NO_3^-	6.07 x 10^{-2}	314
Cl^-	6.39 x 10^{-2}	330
NO_2^-	1.69 x 10^{-1}	873
SO_4^{2-}	6.64 x 10^{-6}	0.0343
CO_3^{2-}	4.30 x 10^{-5}	0.222

Supplementary Table 6 | TcO_4^- removal efficiency from simulated Hanford LAW melter recycle stream.

Material	Conditions (mL g^{-1})	Removal efficiency (%)	Ref.
NDTB-1	200 (40)	13.0 (44.8)	2
SCU-CPN-1	200	89.5	17
SCU-101	100	75.2	14
SCU-100	200	87	11
PQA-Py-Cl	200	92.6	This work
PQA-*p*NH_2Py-Cl	200	87.8	This work
PQA-*p*N$(Me)_2$Py-Cl	200	94.6	This work

Supplementary Table 7 |Composition of simulated Savannah River Site waste solution.

Component	Concentration [M]
Free NaOH	1.33E+00
Total $NaNO_3$	2.60E+00
$NaAl(OH)_4$	4.29E-01
$NaNO_2$	1.34E-01
Na_2SO_4	5.21E-01
Na_2CO_3	2.60E-02
Total Na	5.6
NH_4TcO_4	7.92E-05

Supplementary Table 8 | TcO_4^- removal efficiency from simulated Savannah River Site waste solution.

Material	Conditions (mL g^{-1})	Removal efficiency (%)
PQA-Py-Cl	100	74.6
PQA-*p*NH_2Py-Cl	100	64.2
PQA-*p*$N(Me)_2$Py-Cl	100	76.8

Supplementary Methods

Material synthesis

Synthesis of porous ion exchange material constructed by 1-methylpyridinium chloride moieties (PQA-Py-I).

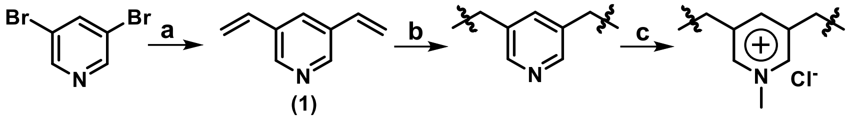

Reagent: (a) potassium vinyltrifluoroborate, $Pd(PPh_3)_4$; (b) AIBN; (c) CH_3I, NaCl

3,5-divinylpyridine (1): 3,5-dibromopyridine (3.0 g, 12.7 mmol), potassium vinyltrifluoroborate (4.06 g, 30.4 mmol), K_2CO_3 (5 g, 36.2 mmol), and $Pd(PPh_3)_4$ (0.37 g, 0.315 mmol) were dissolved in a mixture of toluene (25 mL), THF (25 mL), and H_2O (10 mL), and the resulting mixture was refluxed at 90 °C under N_2 atmosphere for 48 h. The product was extracted with ethyl acetate, washed with brine, dried over Na_2SO_4, and evaporated under reduced pressure, giving the crude compound which was then purified by flash chromatography with hexane/ethyl acetate (5:1) and 1%v/v triethylamine as eluent to afford the title compound as a colorless liquid. Yield: 1.44 g (86%). ^{1}H NMR (400 MHz, $CDCl_3$, 298K, TMS): δ 8.50 (d, 2H, *J*=1.2 Hz), 7.73 (s, 1H), 6.68-6.74 (m, 2H), 5.85 (d, 2H, *J*=14 Hz), 5.40 (d, 2H, *J*=8.8 Hz) ppm. ^{13}C NMR (125 MHz, $CDCl_3$, 298K, TMS) 147.35, 133.35, 132.81, 129.43, 116.46 ppm.

Synthesis of porous polymer constructed by pyridine moieties (POP-Py): **1** (1.0 g) was dissolved in DMF (10 mL), followed by the addition of free radical initiator azobisisobutyronitrile (AIBN, 25 mg). After stirring at room temperature to achieve homogeneity, the mixture was transferred into a 20 mL autoclave and maintained at 100 °C for 24 h. A white solid product (1.0 g, 100% yield) was obtained by extracting the DMF solvent with EtOH and drying in vacuum at 50 °C for 12 h.

Synthesis of porous ion exchange material constructed by 1-methylpyridinium chloride (PQA-Py-Cl): POP-Py (0.5 g) was swelled in acetonitrile (40 mL), followed by addition of iodomethane (1 g). The mixture was then stirred and heated to 80 °C for 48 h. The resulting powder was washed with ethanol and then exchanged with 1 M NaCl solution three times to afford the title product as a light yellow powder.

Synthesis of porous ion exchange material constructed by 4-amino-1-methylpyridinium chloride moieties (PQA-pNH$_2$Py-Cl).

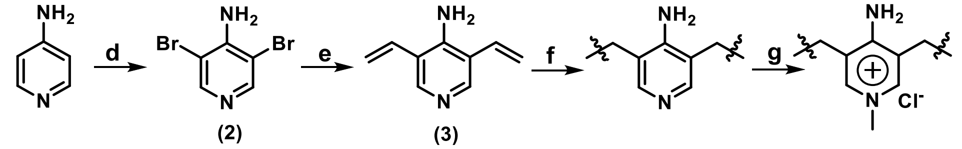

Reagent: (d) N-bromosuccinimide; (e) potassium vinyltrifluoroborate, $Pd(PPh_3)_4$; (f) AIBN; (g) CH_3I, NaCl

3,5-dibromopyridin-4-amine (2): To a mixture of 4-aminopyridine (1.88 g, 20 mmol) in carbon

tetrachloride (80 mL), *N*-bromosuccinimide (7.12 g, 40 mmol) was added in portions. After stirring at room temperature for 24 h, the product was extracted with chloroform, washed with brine, dried over Na_2SO_4, and evaporated under reduced pressure, giving the crude compound which was then purified by flash chromatography with hexane/ethyl acetate (2:1) and 1%v/v triethylamine as eluent to afford the title compound as a yellow powder. Yield: 4.6 g (92%). ^{1}H NMR (400 MHz, DMSO-d6, 298K, TMS): δ 8.21 (s, 2H), 6.40 (s, 2H) ppm.

3,5-divinylpyridin-4-amine (3): 3,5-dibromopyridin-4-amine (3.0 g, 12 mmol), potassium vinyltrifluoroborate (3.96 g, 28.8 mmol), K_2CO_3 (4.8 g, 36 mmol), and $Pd(PPh_3)_4$ (0.348 g, 0.3 mmol) were dissolved in a mixture of toluene (25 mL), THF (25 mL), and H_2O (10 mL), and the resulting mixture was refluxed at 90 °C under N_2 atmosphere for 48 h. The product was extracted with ethyl acetate, washed with brine, dried over Na_2SO_4, and evaporated under reduced pressure, giving the crude compound which was then purified by flash chromatography with hexane/ethyl acetate (5:1) and 1%v/v triethylamine as eluent to afford the title compound as a yellow powder. Yield: 1.56 g (89%). ^{1}H NMR (400 MHz, DMSO-d6, 298K, TMS): δ 8.11 (s, 2H), 6.81-6.88 (m, 2H), 5.96 (s, 2H), 5.59-5.64 (m, 2H), 5.19-5.22 (m, 2H) ppm.

Synthesis of porous polymer constructed by 4-aminepyridine moieties (POP-pNH$_2$Py): **3** (1.0 g) was dissolved in DMF (10 mL), followed by the addition of AIBN (25 mg). After stirring at room temperature to achieve homogeneity, the mixture was transferred into a 20 mL autoclave and maintained at 100 °C for 24 h. A yellow solid product was obtained by extracting the DMF solvent with EtOH and drying in vacuum at 50 °C for 12 h.

Synthesis of porous ion exchange material constructed by 4-amino-1-methylpyridinium chloride moieties (PQA-pNH$_2$Py-Cl): POP-*p*NH$_2$Py (0.5 g) was swelled in acetonitrile (40 mL), followed by the addition of iodomethane (1 g). The mixture was then stirred and heated to 80 °C for 48 h. The resulting powder was washed with ethanol and then exchanged with 1 M NaCl solution three times to afford the title product as a yellow powder.

Synthesis of porous ion exchange material constructed by 4-amino-1-methylpyridinium chloride moieties [PQA-pN(Me)$_2$Py-Cl].

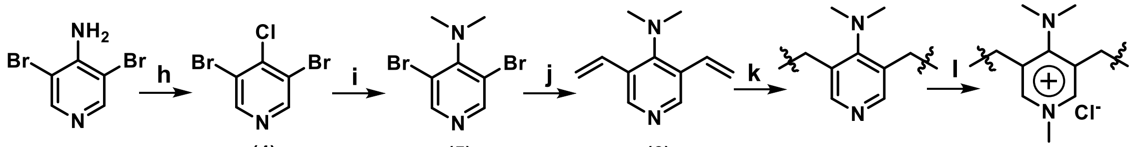

(4) (5) (6)

Reagent: (h) HCl, $NaNO_2$; (i) dimethylamine; (j) potassium vinyltrifluoroborate, $Pd(PPh_3)_4$; (k) AIBN; (i) CH_3I, NaCl

3,5-dibromo-4-chloropyridine (4): 3,5-dibromopyridin-4-amine (4.50 g, 18 mmol) was stirred in concentrated HCl (700 mL) with sodium nitrite (4.5 g, 18 mmol) added in portions at 0-5 °C. After 1 h the temperature was increased to room temperature and stirred for 2 h. Following, 40 wt.% sodium hydroxide was added dropwise, with the temperature kept below 50 °C using an ice bath. After the

reaction, the mixture was extracted with ethyl acetate, dried with K_2CO_3, and evaporated under vacuum to give the title product as white needles. Yield: 4.37 g (90%). ^{1}H NMR (400 MHz, DMSO-d6, 298K, TMS): δ 8.79 (s, 2H) ppm.

3,5-dibromo(pyridine-4-yl)dimethylamine (5): **4** (2.69 g, 10 mmol) and 33 wt.% aqueous solution of dimethylamine (27 mL, 22 mmol), was heated in a Schlenk tube at 118 °C for 20 h. The reaction mixture was then cooled to room temperature, extracted with ethyl acetate, and washed with 1 M K_2CO_3 and water. The organic layer was dried with $MgSO_4$ and evaporated under vacuum to give a pale yellow product. Yield: 4.37 g (90%). ^{1}H NMR (400 MHz, $CDCl_3$, 298K, TMS): δ 8.47 (s, 2H), 3.02 (s, 6H) ppm.

3,5-divinyl(pyridine-4-yl)dimethylamine (6): **5** (3.0 g, 11.8 mmol), potassium vinyltrifluoroborate (3.96 g, 28.8 mmol), K_2CO_3 (4.8 g, 36 mmol), and $Pd(PPh_3)_4$ (0.348 g, 0.3 mmol) were dissolved in a mixture of toluene (25 mL), THF (25 mL), and H_2O (10 mL), and the resulting mixture was refluxed at 90 °C under N_2 atmosphere for 48 h. The product was extracted with ethyl acetate, washed with brine, dried over Na_2SO_4, and evaporated under reduced pressure, giving the crude compound which was then purified by flash chromatography with hexane/ethyl acetate (5:1) and 1%v/v triethylamine as eluent to afford the title compound as a yellow powder. Yield: 1.44 g (86%). ^{1}H NMR (400 MHz, $CDCl_3$, 298K, TMS): δ 8.36 (s, 2H), 6.79-6.86 (m, 2H), 5.66-5.71 (m, 2H), 5.33-5.36 (m, 2H), 2.88 (s, 6H) ppm. ^{13}C NMR (125 MHz, $CDCl_3$, 298K, TMS) 154.33, 148.68, 133.40, 129.04, 115.99, 44.04 ppm.

Synthesis of porous polymer constructed by dimethylaminopyridine [POP-pN(Me)$_2$Py]: **6** (1.0 g) was dissolved in DMF (10 mL), followed by the addition of free radical initiator azobisisobutyronitrile (AIBN, 0.025 g). After stirring at room temperature to achieve homogeneity, the mixture was transferred into a 20 mL autoclave and maintained at 100 °C for 24 h. A yellow solid product was obtained by extracting the DMF solvent with EtOH and drying in vacuum at 50 °C for 12 h.

Synthesis of porous ion exchange material constructed by 4-(dimethylamino)-1-methylpyridin-1-ium moieties [PQA-pN(Me)$_2$Py-Cl]: POP-*p*N(Me)$_2$Py (0.5 g) was swelled in acetonitrile (40 mL), followed by the addition of iodomethane (1 g). The mixture was then stirred and heated to 80 °C for 48 h. The resulting powder was washed with ethanol and then exchanged with 1 M NaCl solution three times to afford the title product as a yellow powder.

Sorption Experiments

Caution! Although Tc-99 is a low-energy β-emitter ($t_{1/2} = 2.13\times10^5$ a), it still possesses significant health risks when inhaled or digested. Standard precautions and procedures for handling radioactive materials should be followed, and all Tc-99 studies were conducted in a licensed laboratory dedicated to radiological investigations.

The aqueous solutions with different rhenium concentrations were obtained by diluting the stock $KReO_4$ solution with the proper amount of distilled water unless otherwise indicated. The pH levels of the solutions were adjusted by HNO_3 or NaOH aqueous solution. The concentrations of uranium during all the experiments were detected by inductively coupled plasma-optical emission spectroscopy (ICP-OES) and inductively coupled plasma-mass spectrometry (ICP-MS) for extra low concentrations. All the adsorption experiments were performed at ambient conditions.

Rhenium sorption isotherms. To obtain adsorption isotherms for PQA-Py-Cl, PQA-*p*NH_2Py-Cl, and PQA-*p*N$(Me)_2$Py-Cl, 5 mg of adsorbent was placed in 10 mL aqueous solutions of varying rhenium concentrations (25-800 ppm). The solutions were then stirred overnight to achieve equilibrium. The solutions were filtered through a 0.45 μm membrane filter and the filtrate was analyzed via ICP to determine the residual rhenium species concentrations. The amount adsorbed or ion-exchange capacity, q_e (mg g^{-1}), at equilibrium was calculated using **Equation 1.**

$$q_e = \frac{(C_0 - C_e)\times V}{m} \qquad (1)$$

Where C_0 and C_e are the initial and equilibrium concentrations, respectively, V is the volume of solution used (mL), and m is the mass of adsorbent (g).

Rhenium sorption kinetics. 16 mg of PQA-Py-Cl, PQA-*p*NH_2Py-Cl, or PQA-*p*N$(Me)_2$Py-Cl was added to an Erlenmeyer flask containing 400 mL of a 50 ppm rhenium aqueous solution. The mixture was then stirred. At increasing time intervals 3 mL aliquots were removed from the mixture, filtered through a 0.45 μm membrane filter, and the filtrate was analyzed by ICP for the remaining rhenium concentration. The adsorption capacity at different intervals was calculated using **Equation 2.**

$$\text{Adsorption capacity (mg/g)} = (C_0 - C_t) \times V/m \qquad (2)$$

where V is the volume of the treated solution (mL), m is the amount of used adsorbent (mg), and C_0 and C_t are the initial concentration and the concentration of rhenium at t (min), respectively.

K_d value calculation. The distribution coefficient (K_d) value as used for the determination of the affinity and selectivity of sorbents for ReO_4^-, is given by **Equation 3.**

$$K_d = \left(\frac{C_0 - C_e}{C_e}\right) \times \frac{V}{m} \qquad (3)$$

where V is the volume of the treated solution (mL), m is the amount of adsorbent (g), C_0 is the initial concentration of rhenium, and C_e is the equilibrium concentration of uranium. In the present work, 10 ppm rhenium aqueous solutions were treated by the various adsorbents overnight at a V/m ratio of 1000 mL g^{-1}.

Rhenium removal from groundwater. Rhenium spiked groundwater sample (50 mL, 1000 ppb) and adsorbents (10 mg) were added to an Erlenmeyer flask with a magnetic stir bar. The mixture was stirred at room temperature. At increasing time intervals 3 mL aliquots were removed from the mixture, filtered through a 0.45 μm membrane filter, and the filtrate was analyzed by ICP-MS for the remaining ReO_4^- concentration. The percentage removal of rhenium species was calculated by **Equation 4.**

$$\text{Removal percentage } (\%) = \frac{C_0 - C_t}{C_0} \times 100 \quad (4)$$

Where C_0 is the initial concentration of rhenium, and C_t is the concentration of rhenium at t (min).

Anion-exchange selectivity against SO_4^{2-} and NO_3^- anions. 5 mg of each adsorbent was weighed into a 20 mL glass vial, and 5 mL of ReO_4^--spiked double distilled water (0.15 mM, 28 ppm) containing different concentrations of $NaNO_3$ (0.15 mM, 1.5 mM, 15 mM, 150 mM, and 450 mM) or Na_2SO_4 (0.015 mM, 0.15 mM, 1.5 mM, 15 mM, 150 mM, and 900 mM) was delivered to the sample vial. After being stirred at room temperature for 24 h, the solutions were filtered through a 0.45 μm membrane filter and the filtrate was analyzed via ICP-OES or ICP-MS to determine the residual rhenium species concentrations.

PQA-pN(Me)$_2$Py-Cl recyclability test. The rhenium species included PQA-*p*N(Me)$_2$Py-Cl [Re@PQA-*p*N(Me)$_2$Py-Cl, ca. 50 mg] was stirred in saturated NaCl aqueous solution (200 mL) for 12 h. The solid was collected by centrifugation and repeated ion exchange with NaCl aqueous solution was performed for another two times. After that, the solid was washed with deionized water until the filtrate was Cl^- free and the solid was dried under vacuum to produce PQA-*p*N(Me)$_2$Py-Cl.

Radiation-resistance measurements. The β-ray and the γ-ray used was provided by an electron accelerator equipped with an electron beam (10 MeV) and a ^{60}Co irradiation source (92.42 PBq), respectively. A β-ray irradiation experiment was conducted by irradiating PQA-*p*N(Me)$_2$Py-Cl at a dose rate of 50 kGy h^{-1} for three different doses (400, 800, and 1000 kGy). A γ-ray irradiation experiment was performed by irradiating PQA-*p*N(Me)$_2$Py-Cl at a dose rate of 3.125 kGy h^{-1} for three different doses (400, 800, and 1000 kGy). The radiation-resistance of PQA-*p*N(Me)$_2$Py-Cl was characterized by FT-IR spectroscopy and further checked by ReO_4^- uptake capacity of the irradiated samples.

Technetium removal from distilled water. TcO_4^- spiked groundwater sample (10 mL, 14.3 ppm based on technetium species) and adsorbents (10 mg) were added to a glass vial with a magnetic stir bar. The mixture was stirred at room temperature. At increasing time intervals aliquots were taken out from the mixture and the remaining TcO_4^- concentration was analyzed by a liquid scintillation counting (LSC) system (Perkin Elmer Quantulus 1220). The percentage removal of technetium species was calculated by **Equation 4.**

Exchange experiments with simulated wastes. The simulated Hanford Low Activity Waste (LAW) Melter Recycle Stream and the simulated Savannah waste solution were prepared according to a

reported protocol (Supplementary reference 17). A certain amount of PQA-*p*N(Me)$_2$Py-Cl was added to 10 mL of the above simulated wastes at V/m values of 200 mL g^{-1} and 100 mL g^{-1}, respectively. After being stirred for 12 h, the suspension was separated with a 0.22 µm nylon membrane filter for LSC analysis.

Computational Methods

Since the polymer is cross-linked and not dissolved in water, we treat it as a separate phase which contains one anion binding site per monomer. The monomer formula weights are 181.07 g mol^{-1}, 196.08 g mol^{-1}, and 224.11 g mol^{-1}. The fraction of binding sites occupied by anion X$^-$ is computed as the ratio, $\theta_X = n_{X\cdot S}/n_{S,\text{total}}$, of polymer sites bound to X$^-$ to total polymer sites, $n_{S,\text{total}}$. Assuming the polymer phase occupies a negligible fraction of the total solution volume, the occupancy fraction is also related to the solution concentration of ions through mass balance. Mass balance states that the formal concentration of X$^-$ ions is

(5) $$\frac{n_{X,\text{total}}}{V_\text{total}} = f_X = [X^-] + [S]\theta_X$$

where $[X^-]$ is the concentration of anions in the aqueous phase and $[S] = \frac{n_{S,\text{total}}}{V_\text{total}}$ is the formal concentration of polymer binding sites. Finally, the equilibrium constant for the anion exchange reaction,

(6) $$S^+ \cdot Cl^- + X^-_{(aq)} \xrightarrow{\Delta\Delta G_\text{exch}} S^+ \cdot X^- + Cl^-_{(aq)}$$

is

(7) $$K_{\text{Cl}^- \to X^-} = \frac{\theta_{X^-}\gamma_{\text{Cl}^-}[\text{Cl}^-]}{\theta_{\text{Cl}^-}\gamma_{X^-}[X^-]} \approx \frac{[\text{Cl}^-](f_{X^-} - [X^-])}{[X^-](f_{\text{Cl}^-} - [\text{Cl}^-])} .$$

The approximation assumes the two aqueous Debye-Huckel ionic activity coefficients are similar so $\frac{\gamma_{\text{Cl}^-}}{\gamma_{X^-}} = 1$ (appropriate for ions of the same charge and hydrated radius). For isotherms where all Cl$^-$ comes from the polymer, f_{Cl^-} = [S] and the equation for the isotherm is:

(8) $$\rho_S\theta_X = f_{X^-} - [X^-] = \frac{K_{\text{Cl}^- \to X^-}}{2}\left(\sqrt{[X^-]^2 + 4[X^-][S]/K_{\text{Cl}^- \to X^-}} - [X^-]\right)$$

However, a more direct way to plot the isotherm would be to take the logarithm of **Equation 7** and show log ([X$^-$]/[Cl$^-$]) as the independent variable.

Ion-binding free energies for each polymer were estimated from calculations on the truncated fragments shown in Supplementary Fig. 23. Quantum calculations were carried out using density functional theory with the M06 exchange and correlation functionals to compute the cluster formation free energy,

(9) $$\Delta G^0 = RT \ln \frac{q(S^+X^-)}{q(S^+)q(X^-)}$$

where each q is a partition functions of a gas-phase cluster, defined explicitly in **Equations 12-14**. A 6-311++G(2d,2p) basis was used for all atoms except Tc, which was treated with the def2-tzvp basis and associated empirical core potential. This combination of basis sets and functionals was found previously to reproduce the experimental structure of the pertechnetate anion.[18.19] All our calculations used a "fine" grid setting which targets an energy accuracy of 10^{-7} Hartree – in this case by using Mura-Knowles radial quadrature with 60-141 points per atom and Lebedev angular quadrature with 590-974 points per atom before pruning.

Solvation free energies were computed using the 3D RISM method with the Kovalenko-Hirata closure[20] as implemented by Case and coworkers.[21] We also applied the pressure correction of Supplementary reference 22 as implemented by Misin and coworkers.[23] Solvent interaction Lennard-Jones parameters for all atoms were computed from MMFF94 nonbonded parameters between each atom and water oxygen. These were calibrated so that the parameters would be computed correctly by the Lorentz-Berthelot combination rule employed by the 3D RISM model with its SPC water model. These are summarized later as relative solvation free energies for the complexation reaction,

(10) $$\Delta\Delta G_{\text{solv}} = \Delta G_{\text{solv}}(S^+X^-) - \Delta G_{\text{solv}}(S^+) - \Delta G_{\text{solv}}(X^-)$$

Since water that hydrogen bonds to the side-chain amine may affect the distribution of charge in the ring strongly in the **b** structure (Fig 4, main text), we also computed the binding energies for **c** structure. For each anion, its complex with structure **c** was prepared by copying the position of the water in energy-minimized **c** into the minimized **b**-anion structure in the same position and orientation relative to the NH_2 group. We did not reoptimize these complexes, since re-optimization would result in water positions partially hydrogen bonded to the anions, rather than directly affecting the NH_2 group as intended.

Supplementary Table 4 shows the individual component energies used in the calculation, including quantum-mechanical gas-phase binding constants (ΔG^0) and the free energies of hydration in pure water. Note that the solvation free energy difference from Cl^- to TcO_4^- is 52 kJ mol^-, following the Born trend in Supplementary reference 24. The final three rows tabulate the free energies of anion exchange. The free energy of exchange relative to Cl^- (corresponding to **Equation 6**) is a difference between two half-reactions,

(11) $$\Delta G_{\text{exch}} = \Delta G^0(S^+X^-) + \Delta\Delta G_{\text{solv}} \qquad S^+ + X^-_{(aq)} \xrightarrow{\Delta G_{\text{exch}}} S^+ \cdot X^-$$

The trends show that all structures are selective for TcO_4^- over NO_3^- and HSO_4^-. We did not compare directly to SO_4^{2-} because the difference in net charge would introduce long-range correction terms,

incurring additional numerical error, and a dependence on solution ionic strength. However, the polymer environment is relatively hydrophobic, and it may be the case that SO_4^{2-} is deprotonated during exchange. On the other hand, if the pKa of HSO_4^- is identical in both the polymer and aqueous phases, then the free energy of exchanging HSO_4^- will also equal the free energy of exchanging SO_4^{2-}.

Gas-phase partition coefficients were calculated considering only the anion translational and rotational contributions using:

(12) $$\frac{q(S^+X^-)}{q(S^+)q(X^-)} \approx \frac{q_{vib}(X^- \,|S^+)}{q_{tr}(X^-)q_{rot}(X^-)} e^{-\beta(E_{S^+X^-} - E_{S^+} - E_{X^-})}$$

With energies, *E,* computed from the ground state energy of each molecule and $q_{tr}(X^-)q_{rot}(X^-)$ the standard expressions for ligand gas-phase translational and rotational partition functions including symmetry numbers (1 for Cl^-, 2 for HSO_4^-, and 12 for NO_3^- and TcO_4^-). **Equation 12** is appropriate if the polymer and anion behave as rigid bodies during binding, so their internal degrees of freedom are frozen. The numerator treats the anion as a rigid body with 6 (or 3 for Cl^-) vibrational degrees of freedom,

(13) $$q_{vib}(X^- \,|S^+) = \prod_{j=1}^{6} \frac{e^{-\beta h \nu_j/2}}{1 - e^{-\beta h \nu_j}} \quad .$$

Here ν_j are the vibrational frequencies of external coordinates for X^- in the field of S^+ obtained by diagonalizing the mass-weighted projected Hessian matrix, $\mathrm{diag}(M, I)^{-1/2}\,[T|R]^T H\,[T|R]\,\mathrm{diag}(M, I)^{-1/2}$, with H the Hessian for the anion coordinates in the presence of S^+, M the total mass of X^- (scaling translations, *T*), I the principle moments of inertia for X^- (scaling infinitesimal rotations, *R*). The 3N by 3 matrices *T* and *R* are given explicitly by,

(14) $$T_{ij} = \begin{cases} 1, i \bmod 3 = j \\ 0, \text{otherwise} \end{cases}, \qquad R_{ij} = r_{\lfloor i/3 \rfloor} \times L_{i \bmod 3}$$

with r the vector of anion coordinates and L_j a unit vector in the direction of the jth moment of inertia (I_j). For X = Cl^-, the rotational modes are omitted.

Supplementary Reference

1. Li, Y., Wang, Q., Li, Q., Zhang, Z., Zhang, L. & Liu, X. Simultaneous speciation of inorganic rhenium and molybdenum in the industrial wastewater by amino-functionalized nano-SiO_2. *J. Taiwan. Inst. Chem. E.* **55**, 126-132 (2015).
2. Wang, S., Yu, P., Purse, B. A., Orta, M. J., Diwu, J., Casey, W. H., Phillips, B. L., Alekseev, E. V., Depmeier, W., Hobbs, D. T. & Albrecht-Schmitt, T. E. Selectivity, kinetics, and efficiency of reversible anion exchange with TcO_4^- in a supertetrahedral cationic framework. *Adv. Funct. Mater.* **22**, 2241−2250 (2012).
3. Gao, Y., Chen, K., Tan, X., Wang, X., Alsaedi, A., Hayat, T. & Chen, C. Interaction mechanism of Re(VII) with zirconium dioxidenanoparticles archored onto reduced graphene oxides. *ACS Sustainable Chem. Eng.* **5**, 2163-2171 (2017).
4. Cui, X., Zhang, P., Wang, Y., Lou, Z. & Shan, W. Improving Re(VII) adsorption on diisobutylamine-functionalized graphene oxide. *ACS Sustainable Chem. Eng.* **5**, 1010-1018 (2017).
5. Zu, J. H., Wei, Y. Z., Ye, M. S., Tang, F. D., He, L. F. & Liu, R. Q. Preparation of a new anion exchanger by pre-irradiation grafting technique and its adsorptive removal of rhenium (VII) as analogue to ^{99}Tc. *Nucl. Sci. Tech.* **26**, 69-75 (2015).
6. Zu, J., Ye, M., Wang, P., Tang, F. & He, L. Design of a strong-base anion exchanger and its adsorption and elution behavior for rhenium(VII). *RSC Adv.* **6**, 18868-18873 (2016).
7. Shu, Z. & Yang, M. Adsorption of rhenium(VII) with anion exchange resin D318. *Chinese. J. Chem. Eng.* **18**, 372-376 (2010).
8. Xiong, C., Yao, C. & Wu, X. Adsorption of rhenium(VII) on 4-amino-1,2,4-triazole resin. *Hydrometallurgy* **90**, 221-226 (2008).
9. Jia, M., Cui, H., Jin, W., Zhu, L., Liu, Y. & Chen, J. Adsorption and separation of rhenium(VII) using N-methylimidazolium functionalized strong basic anion exchange resin. *J. Chem. Technol. Biot.* **88**, 437-443 (2013).
10. Zhu, L., Xiao, C., Dai, X., Li, J., Gui, D., Sheng, D., Chen, L., Zhou, R., Chai, Z., Albrecht-Schmitt, T. E. & Wang, S. Exceptional perrhenate/pertechnetate uptake and subsequent immobilization by a low-dimensional cationic coordination polymer: overcoming the hofmeister bias selectivity. *Environ. Sci. Technol. Lett.* **4**, 316-322 (2017).
11. Sheng, D., Zhu, L., Xu, C., Xiao, C., Wang, Y., Wang, Y., Chen, L., Diwu, J., Chen, J., Chai, Z., Albrecht-Schmitt, T. E. & Wang, S. Efficient and selective uptake of TcO_4^- by a cationic metal–organic framework material with open Ag^+ sites. *Environ. Sci. Technol.* **51**, 3471-3479 (2017).
12. Fei, H., Rogow, D. L. & Oliver, S. R. Reversible anion exchange and catalytic properties of two cationic metal-organic frameworks based on Cu(I) and Ag(I). *J. Am. Chem. Soc.* **132**, 7202-7209 (2010).
13. Banerjee, D., Xu, W., Nie, Z., Johnson, L. E. V., Coghlan, C., Sushko, M. L., Kim, D., Schweiger, M. J., Kruger, A. A., Doonan, C. J. & Thallapally, P. K. Zirconium-based metal-organic framework for removal of perrhenate from water. *Inorg. Chem.* **55**, 8241-8243 (2016).
14. Zhu, L., Sheng, D., Xu, C., Dai, X., Silver, M. A., Li, J., Li, P., Wang, D., Wang, Y., Chen, L., Xiao, C., Chen, J., Zhou, R., Zhang, C., Farha, O. K., Chai, Z., Albrecht-Schmitt, T. E. & Wang, S.

Identifying the recognition site for selective trapping of $^{99}TcO_4^-$ in a hydrolytically stable and radiation resistant cationic metal–organic framework. *J. Am. Chem. Soc.* **139**, 14873-14876 (2017).
15. Drout, R. J., Otake, K., Howarth, A. J., Islamoglu, T., Zhu, L., Xiao, C., Wang, S. & Farha, O. K. Efficient capture of perrhenate and pertechnetate by a mesoporous Zr metal-organic framework and examination of anion binding motifs. *Chem. Mater.* **30**, 1277-1284 (2018).
16. Banerjee, D., Elsaidi, S. K., Aguila, B., Li, B., Kim, D., Schweiger, M. J., Kruger, A. A., Doonan, C. J. & Ma, S., Thallapally, P. K. Removal of pertechnetate-related oxyanions from solution using functionalized hierarchical porous frameworks. *Chem. Eur. J.* **22**, 17581-17584 (2016).
17. Li, J., Dai, X., Zhu, L., Xu, C., Zhang, D., Silver, M. A., Li, P., Chen, L., Li, Y., Zuo, D., Zhang, H., Xiao, C., Chen, J., Diwu, J., Farha, O. K., Albrecht-Schmitt, T. E., Chai, Z. & Wang, S. $^{99}TcO_4^-$ remediation by a cationic polymeric network. *Nat. Commun.* **9**, 3007 (2018).
18. Williams, C. D., Burton, N. A., Travis, K. P. & Harding, J. H. The development of a classical force field to determine the selectivity of an aqueous Fe^{3+}–EDA complex for TcO_4^- and SO_4^{2-}. *J. Chem. Theory Comput.* **10**, 3345-3353 (2014).
19. Weaver, J., Soderquist, C. Z., Washton, N. M., Lipton, A. S., Gassman, P. L., Lukens, W. W., Kruger, A. A., Wall, N. A. & McCloy, J. S. Chemical trends in solid alkali pertechnetates. *Inorg. Chem.* **56** 2533–2544 (2017).
20. Kovalenko, A. & Hirata. F. Self-consistent description of a metal-water interface by the Kohn-Sham density functional theory and the three-dimensional reference interaction site model. *J. Chem. Phys.* **110**, 10095-10112 (1999).
21. Johnson, J., Case, D. A., Yamazaki, T., Gusarov, S., Kovalenko, A. & Luchko. T. Small molecule hydration energy and entropy from 3D-RISM. *J. Phys.: Condens. Matter*. **28**, 344002 (2016).
22. Sergiievskyi, V., Jeanmairet, G., Levesque, M. & Borgis, D. Solvation free-energy pressure corrections in the three dimensional reference interaction site model. *J. Chem. Phys.* **143**, 184116 (2015).
23. Misin, M., Fedorov, M. & Palmer, D. Hydration free energies of ionic species by molecular theory and simulation. *J. Phys. Chem. B.* **120**, 975-983 (2016).
24. Williams, C. D. & Carbone, P. A classical force field for tetrahedral oxyanions developed using hydration properties: The examples of pertechnetate (TcO_4^-) and sulfate (SO_4^{2-}). *J. Chem. Phy.* **143**, 174502 (2015).
